# Semiparametric maximum likelihood probability density estimation

**DOI:** 10.1371/journal.pone.0259111

**Published:** 2021-11-09

**Authors:** Frank Kwasniok

**Affiliations:** Department of Mathematics, University of Exeter, Exeter, United Kingdom; Arizona State University, UNITED STATES

## Abstract

A comprehensive methodology for semiparametric probability density estimation is introduced and explored. The probability density is modelled by sequences of mostly regular or steep exponential families generated by flexible sets of basis functions, possibly including boundary terms. Parameters are estimated by global maximum likelihood without any roughness penalty. A statistically orthogonal formulation of the inference problem and a numerically stable and fast convex optimization algorithm for its solution are presented. Automatic model selection over the type and number of basis functions is performed with the Bayesian information criterion. The methodology can naturally be applied to densities supported on bounded, infinite or semi-infinite domains without boundary bias. Relationships to the truncated moment problem and the moment-constrained maximum entropy principle are discussed and a new theorem on the existence of solutions is contributed. The new technique compares very favourably to kernel density estimation, the diffusion estimator, finite mixture models and local likelihood density estimation across a diverse range of simulation and observation data sets. The semiparametric estimator combines a very small mean integrated squared error with a high degree of smoothness which allows for a robust and reliable detection of the modality of the probability density in terms of the number of modes and bumps.

## 1 Introduction

Estimating a probability density function from a given data sample is one of the oldest and most fundamental smoothing problems in statistics with a vast literature on the underlying theoretical issues and numerous applications in almost all areas of science, for example, in astronomy, bioinformatics, climate science, finance, economics, engineering, geoscience and medicine. Methods for probability density estimation can be classified into three groups: parametric, nonparametric and semiparametric approaches.

In the parametric approach, a small number of parameters are fitted by maximum likelihood or the method of moments within some prescribed model family such as, for example, the Gaussian, gamma, log-normal or beta distribution. Obviously, the flexibility of this approach is limited as the chosen distribution family imposes quite a strong constraint on the range of probability densities which can be covered.

Nonparametric probability density estimation offers a far greater flexibility in modelling a given data sample. The most widely used nonparametric technique is kernel density estimation [1–3]. Any kernel density estimate depends crucially on the choice of bandwidth [1, 4]. Basically, two different data-driven bandwidth selection techniques are available: the classical cross-validation approach [1] and the plug-in method [5]. Until relatively recently the plug-in method was based on the normal reference rule [6] which can adversely affect the density estimate if the normality assumption does not hold very well, particularly for bi- or multimodal densities. An improved plug-in bandwidth selection technique dropping the normal reference rule was proposed and superior practical performance demonstrated on a host of different simulation examples [7].

Standard kernel density estimation with second-order kernels has a couple of limitations. Firstly, in one dimension no positive kernel can have a mean integrated squared error decaying faster than *N*^−4/5^ with *N* being the sample size. In higher dimensions the speed of convergence is even slower. Secondly, kernel density estimates on bounded or semi-infinite domains exhibit considerable boundary bias as the kernel does not take account of information about the support of the density. Thirdly, standard kernels lack local adaptivity which often leads to the occurrence of spurious bumps and a tendency to flatten the peaks and valleys of the density.

Higher-order kernels have been used to improve accuracy (for example, [8, 9]), but these have the drawback of not guaranteeing to provide a proper non-negative density estimate. Boundary bias has been tackled by boundary kernel estimators [10] and boundary correction schemes [11]. The lack of local adaptivity has been addressed by adaptive kernel estimators [12–14]. The most systematic and comprehensive solution attempt to all of the three issues is the diffusion estimator [7] which has improved asymptotic accuracy, deals naturally with domain boundaries, features data-adaptive smoothing and always provides a bona fide probability density, that is, it is non-negative and integrates to unity.

Semiparametric density estimation methodologies lie in between parametric and nonparametric approaches, combining advantages of both. While technically they are parametric the number of parameters and thus the complexity of the model may vary in a data-driven manner, usually increasing when more data are available. Semiparametric density estimates are considerably more flexible than the parametric approach but usually smoother than kernel density estimates. This is particularly advantageous when assessing the number of modes or bumps of a distribution. In the following four different semiparametric approaches are discussed.

Finite mixture models [15] are convex linear combinations of component densities taken from a standard family of distributions. Gaussian mixture models are most common; on bounded or semi-infinite domains also beta, gamma, Weibull or other mixture models are possible. The number of components can be treated as a hyperparameter and determined by cross-validation or with an information criterion. Alternatively, it can be treated as a random variable and estimated in a Bayesian framework.

In local likelihood density estimation [16, 17] the probability density at a given point is estimated by assuming a simple local model for the log-density, typically constant, linear or quadratic, in the neighbourhood of that point and inferring its parameters by maximum likelihood. The domain of influence of the local model is described by a kernel function with a bandwidth parameter. The method always gives a non-negative density estimate but exhibits boundary bias on bounded or semi-infinite intervals.

Another approach is to expand the density into a system of orthogonal functions. The idea goes back to the classical Edgeworth and Gram–Charlier type A series where the density is expanded into Hermite polynomials about a Gaussian reference density and expansion coefficients are determined by moment matching. These classical expansions are usually limited to just a few terms and can cover only densities relatively close to a Gaussian. The approach can be generalised [18, 19] to arbitrary domains of support and boundary conditions by choosing an appropriate set of orthogonal functions, either polynomial or trigonometric, and determining the expansion coefficients by projection of the basis functions onto the data sample which is particularly simple. See [20] for an extensive review. However, the density estimates obtained from a truncated set of basis function are not necessarily non-negative. A similar method which always provides a bona fide density estimate expands a density on a bounded interval into Bernstein polynomials and derives expansion coefficients from a discretised version of the empirical distribution function [21]. For all of these series expansions the inference connects neither to maximum likelihood nor to the method of moments which is somewhat unsatisfactory.

A more principled methodology may be obtained from expanding the logarithm of the density rather than the density itself into a set of basis functions. While being technically more involved this approach automatically yields a bona fide probability density for any truncation of the set of basis functions. The inference is rooted in maximum likelihood estimation in sequences of exponential families which is very well understood and has nice properties such as strict concavity of the log-likelihood function and convergence in relative entropy [22, 23]. The idea first occurred with the Gram–Charlier type C series where the log-density on the infinite domain is expanded into Hermite polynomials. It was later proposed as maximum penalized likelihood density estimation [24–27] where the log-likelihood function is augmented with a roughness penalty functional as regularisation term to enforce smoothness of the density. Log-density estimation without a roughness penalty has been done using polynomials [28], splines [29–31] and wavelets [32] as basis functions. Log-density estimation with polynomial basis functions was used to estimate large-deviation rate functions [33] and was extended to a non-stationary setting in order to detect and predict critical transitions in dynamical systems [34]. Maximum likelihood log-density estimation is equivalent to the maximum entropy method [35–38]. A nonparametric extension of the maximum entropy method was proposed recently [39].

The present paper studies one-dimensional semiparametric log-density estimation from a theoretical as well as numerical point of view. It extends previous work by including the following novel elements:

(i) Bounded, infinite and semi-infinite domains of support of the density are considered.
(ii) The bulk basis functions such as polynomial, spline and trigonometric [23] are considered at the same time; systematic and automatic model selection over the type and number of basis functions with the Bayesian information criterion is proposed. While not theoretically deep this has to the best knowledge of the author not been done before and it proves very beneficial in terms of practical performance of the estimator.
(iii) Linearly extrapolated polynomial basis functions are derived for infinite and semi-infinite domains which have better inference properties than the standard polynomials.
(iv) The spline basis functions are here not restricted to natural splines; they are more flexible as they have no curvature condition at the endpoints. Moreover, novel knot placement and deletion schemes are proposed.
(v) The bulk basis functions are augmented with logarithmic or rational boundary terms which increases the efficiency of the method for domains with boundaries. For example, logarithmic terms allow to capture power-law decay to zero of the probability density at a boundary point.
(vi) A statistically orthogonal formulation of the inference problem is introduced based on orthogonalisation of the basis functions with respect to a data-adaptive scalar product. While the use of classical orthogonal polynomials such as Legendre polynomials [28] or *B*-splines [29, 31] is standard in log-density estimation here basis functions are constructed which are exactly orthogonal over the data set under consideration providing a very good condition of the optimization problem close to the solution even for rather high-dimensional models.
(vii) A comprehensive comparison of the new method with standard methods such as kernel density estimation and finite mixture models is performed.

The remainder of the paper is organised as follows: In Section 2 the general methodology is developed. Bounded, infinite and semi-infinite domains of support are discussed in detail in Sections 3, 4 and 5. Section 6 explains the algorithm for maximizing the likelihood function; Section 7 details the model selection procedure. The results are presented and discussed in Section 8. Some conclusions are drawn in Section 9.

## 2 Semiparametric log-density estimation

We address the problem of estimating the univariate probability density function *f*_*X*_(*x*) of the random variable *X* from a given data sample. The domain of support of the probability density, *D*_*X*_, may be bounded (*D*_*X*_ = [*a*, *b*]), infinite (*D*_*X*_ = (−∞, ∞)) or semi-infinite (*D*_*X*_ = [*a*, ∞)). Often it is numerically convenient to introduce a linear rescaling *Y* = (*X* − *c*)/*s* and *y* = (*x* − *c*)/*s* with suitable constants *c* and *s* > 0. The domain *D*_*X*_ is mapped onto a domain *D*_*Y*_. The scaled probability density *f*_*Y*_(*y*) is linked to *f*_*X*_(*x*) by *f*_*Y*_((*x* − *c*)/*s*) = *sf*_*X*_(*x*). In the following, for ease of notation, the subscript *Y* when referring to quantities associated with the rescaled variable *Y* will be dropped and only the subscript *X* will be kept when referring to quantities associated with the original variable *X*.

### 2.1 The probability density model

The probability density function is modelled as

$$\begin{array}{c} p\left(y|\alpha \right)=\frac{1}{Z(\alpha )}\text{exp}\left[U\left(y|\alpha \right)\right]=\text{exp}\left[V\left(y|\alpha \right)\right] \end{array}$$ (1)

where the *potential function U* is expanded as

$$\begin{array}{c} U\left(y|\alpha \right)=\psi \left(y\right)+\sum_{j=1}^{J}\alpha_{j}\phi_{j}\left(y\right) \end{array}$$ (2)

with a given function *ψ*, a set of prescribed basis functions {*ϕ*_1_, …, *ϕ*_*J*_} and expansion coefficients ***α*** = (*α*_1_, …, *α*_*J*_)^T^. The *normalization constant* or *partition function* is given by

$$\begin{array}{c} Z\left(\alpha \right)=\int_{D}\text{exp}\left[U\left(y|\alpha \right)\right]\;dy. \end{array}$$ (3)

The potential function of the normalised probability density is

$$\begin{array}{c} V(y|\alpha )=U(y|\alpha )-A(\alpha ) \end{array}$$ (4)

with the *log-partition function*

$$\begin{array}{c} A(\alpha )=\text{log}\;Z(\alpha ). \end{array}$$ (5)

For a well-specified and non-redundant model we require the set of functions {1, *ψ*, *ϕ*_1_, …, *ϕ*_*J*_} to be linearly independent on *D*. The potential function *U* is only determined up to an arbitrary additive constant.

The probability density model is a Gibbs or Boltzmann distribution. In statistical terms, it is an *exponential family* [40–42] of order *J* in *canonical form* with *canonical parameters* ***α*** = (*α*_1_, …, *α*_*J*_)^T^ and *canonical sufficient statistics* *ϕ*(*y*) = (*ϕ*_1_(*y*), …, *ϕ*_*J*_(*y*))^T^. The exponential family is *minimal* and *full*. The *canonical parameter space* $P=\{\alpha \in R^{J}\;|\;Z\left(\alpha \right)<\infty \}$ is a convex subset of $R^{J}$. The exponential family is called *regular* if P is an open set. The cumulant generating function of the sufficient statistics is *K*(**t**|***α***) = *A*(***α*** + **t**) − *A*(***α***).

The choice of basis functions is very flexible which makes the modelling framework powerful. Depending on the support of the density and the nature of the data to be investigated the basis functions could be polynomials, spline functions, trigonometric functions or wavelets, possibly augmented with logarithmic or rational boundary terms.

The *base measure* of the exponential family is *h*(*y*) = exp[*ψ*(*y*)]. We here mostly have *ψ*(*y*) = 0 but the option allows to include into the modelling framework some well-known distributions such as the Rayleigh distribution (*U*(*y*) = log *y* + *α*_1_ *y*^2^), the Weibull distribution with known shape parameter (*U*(*y*) = (*k* − 1) log *y* + *α*_1_ *y*^*k*^) or the inverse Gaussian distribution (*U*(*y*) = −(3/2) log *y* + *α*_1_(1/*y*) + *α*_2_ *y*). Another application could be a choice *ψ*(*y*) = log Π(*y*) = log *s* + log Π_*X*_(*c* + *sy*) where Π_*X*_(*x*) is some pilot probability density of *X* obtained from the data, for example, via kernel density estimation which is then post-processed or refined by the exponential family model. This was proposed in the literature [43] and Poisson regression used for the inference. As such the present work contributes a new algorithm for this type of problem but we will not pursue this here.

Ideally, the model class for the probability densities of *X* − *c* and *Y* should be the same, that is, there is a bijective mapping between a parameter vector ***α*** and a parameter vector $\tilde{\alpha }$ such that $C+\psi \left(sy\right)+\sum_{j}{\tilde{\alpha }}_{j}\phi_{j}\left(sy\right)=\psi \left(y\right)+\sum_{j}\alpha_{j}\phi_{j}\left(y\right)$ with a constant *C* which may depend on ***α***. Then maximum likelihood estimation for *p*(*y*) is equivalent to maximum likelihood estimation directly for *p*_*X*_(*x*). This invariance of the exponential family under linear scaling is satisfied in all of the models presented here.

### 2.2 Parameter estimation

Given a data sample of size *N*, {*x*_1_, …, *x*_*N*_}, the data points are rescaled as *y*_*n*_ = (*x*_*n*_ − *c*)/*s*; *n* = 1, …, *N*. The sample mean of any function *e*(*y*) on *D* is introduced as

$$\begin{array}{c} \left\langle e\right\rangle =\frac{1}{N}\sum_{n=1}^{N}e\left(y_{n}\right). \end{array}$$ (6)

The log-likelihood function is

$$\begin{array}{c} l=N(\langle U\rangle -\text{log}\;Z); \end{array}$$ (7)

the log-likelihood function of the original data sample is *l*_*X*_ = *l* − *N* log *s*. In the interior of the parameter space, α∈intP, the population means of the basis functions, **m** = (*m*_1_, …, *m*_*J*_)^T^, are

$$\begin{array}{c} m_{j}=E_{\alpha }\left(\phi_{j}\right)=\int_{D}\phi_{j}\left(y\right)\text{exp}\left[V\left(y\right)\right]\;dy=\frac{\partial A}{\partial \alpha_{j}}; \end{array}$$ (8)

the second population moments are

$$\begin{array}{c} Q_{jk}=E_{\alpha }\left(\phi_{j}\phi_{k}\right)=\int_{D}\phi_{j}\left(y\right)\phi_{k}\left(y\right)\text{exp}\left[V\left(y\right)\right]\;dy=\frac{\partial^{2}A}{\partial \alpha_{j}\partial \alpha_{k}}+\frac{\partial A}{\partial \alpha_{j}}\frac{\partial A}{\partial \alpha_{k}}. \end{array}$$ (9)

The gradient of the log-likelihood function, the score function, ∇_**α**_*l* = **g** = (*g*_1_, …, *g*_*J*_)^T^, is given by

$$\begin{array}{c} g_{j}=\frac{\partial l}{\partial \alpha_{j}}=N\left(\left\langle \phi_{j}\right\rangle -m_{j}\right) \end{array}$$ (10)

and the Hessian matrix of second derivatives **H** is

$$\begin{array}{c} H_{jk}=\frac{\partial^{2}l}{\partial \alpha_{j}\partial \alpha_{k}}=-N\frac{\partial^{2}A}{\partial \alpha_{j}\partial \alpha_{k}}=-N{\text{Cov}}_{\alpha }\left(\phi_{j},\phi_{k}\right)=-N\left(Q_{jk}-m_{j}m_{k}\right). \end{array}$$ (11)

A stationary point of the log-likelihood function, $\hat{\alpha }$, is a zero of the score function and is characterised by the likelihood equations

$$\begin{array}{c} \text{m}\left(\hat{\alpha }\right)=\left\langle \phi \right\rangle . \end{array}$$ (12)

For a minimal and full exponential family the log-partition function is real analytic and strictly convex, the log-likelihood function is strictly concave and the Hessian matrix is negative definite on int P [40–42]. This holds for any set of basis functions and any truncation level *J*. If a solution to the likelihood equations exists it is unique and corresponds to the unique global maximum of the likelihood function. The maximum likelihood estimate of *p* then is $\hat{p}\left(y\right)=\text{exp}\left[U\left(y|\hat{\alpha }\right)-A\left(\hat{\alpha }\right)\right]$ and the maximum likelihood estimate of *f*_*X*_ is

$$\begin{array}{c} {\hat{f}}_{X}\left(x\right)=\text{exp}[U(\frac{x-c}{s}\;\;|\;\hat{\alpha })-\text{log}\;s-A\left(\hat{\alpha }\right)]. \end{array}$$ (13)

Eq (13) constitutes the formal definition of the semiparametric density estimator to which we will refer later.

The likelihood equations have an intuitive interpretation. At the solution the population means of the basis functions match the sample means. For polynomial basis functions maximum likelihood estimation is identical to the classical method of moments; for other basis functions it corresponds to an extended method of moments with general moment functions.

The question of existence of the maximum likelihood estimator is linked to the *regularity* or *steepness* of the exponential family. For regular or steep exponential families the maximum likelihood estimator exists for any generic data sample. Almost all models considered here, in particular the most relevant ones in practice, are regular. This includes all models generated by (linearly extrapolated) polynomials and splines as well as trigonometric functions, possibly augmented with logarithmic boundary terms, on bounded, infinite and semi-infinite domains. See Sections 3, 4 and 5 for details on the basis functions for the different domains. A detailed discussion of the existence of the maximum likelihood estimator is given in S1 Appendix.

There are relationships between maximum likelihood estimation in exponential families and the *truncated moment problem* [44–46] as well as the *moment-constrained maximum entropy principle* [35, 47, 48]. These relationships are not new, yet they appear to have not been fully exploited as the different problems are studied in disjoint scientific communities. In statistics, exponential families generated by polynomials are rarely discussed beyond the normal, exponential and truncated normal distributions; on the other hand, the non-statistical community seems to be often unaware of the statistical link. The different problems provide complementary points of view on the existence of solutions. A discussion is given in S2 Appendix. In particular the statements in Theorem 2 and subsequently Theorem 3 therein have, to the best knowledge of the author, not been given in the literature before. The proposition is that the conditions of positive definiteness of the relevant Hankel matrices which in most settings guarantee the existence of a unique moment-constrained maximum entropy solution are always satisfied provided that the moment estimates are given as sample moments of a generic data sample. The proposition equally holds for bounded, infinite and semi-infinite intervals. Note that here we are using newly introduced linearly extrapolated polynomials rather than standard polynomials for infinite and semi-infinite domains because of their superior inference properties (see Subsections 4.1 and 5.1).

### 2.3 Parameter uncertainty

The expected Fisher information matrix for exponential families is equal to the observed Fisher information matrix and both are given as

$$\begin{array}{c} J=-\hat{H}=N[E_{\hat{\alpha }}\left(\phi \phi^{T}\right)-E_{\hat{\alpha }}\left(\phi \right)E_{\hat{\alpha }}\left(\phi^{T}\right)] \end{array}$$ (14)

where $\hat{H}$ denotes the Hessian of the log-likelihood at the maximum likelihood solution. Parameter errors are asymptotically normal with zero mean and covariance matrix **C** = **J**^−1^.

For dependent data, for example, if the data sample comes as a time series with temporal correlation we use an error covariance inflation technique [49]. The general expression for the error covariance matrix is

$$\begin{array}{c} C=J^{-1}VJ^{-1} \end{array}$$ (15)

with $V=E_{\hat{\alpha }}\left(gg^{T}\right)$. In the case of independent data we have **V** = **J** and recover the result above. The contribution of data point *y*_*n*_ to the log-likelihood is *l*_*n*_ = *U*(*y*_*n*_) − log *Z* and we have $\nabla_{\alpha }l_{n}{|}_{\alpha =\hat{\alpha }}=\phi \left(y_{n}\right)-\langle \phi \rangle$. A block length $l_{b}$ is chosen such that data in disjoint blocks of *l*_b_ consecutive data points can be considered independent. We assume that *N* is an integer multiple of *l*_b_. The variables

$$\begin{array}{c} {\bar{g}}_{i}=\sum_{n=\left(i-1\right)l_{b}+1}^{il_{b}}\left[\phi \left(y_{n}\right)-\left\langle \phi \right\rangle \right] \end{array}$$ (16)

are formed for *i* = 1, …, *N*/*l*_b_. The covariance matrix of ${\bar{g}}_{i}$ is estimated empirically from the sample ${\left\{{\bar{g}}_{i}\right\}}_{i=1}^{N/l_{b}}$ and we obtain

$$\begin{array}{c} V=\sum_{i=1}^{N/l_{b}}E_{\hat{\alpha }}\left({\bar{g}}_{i}{\bar{g}}_{i}^{T}\right)=\sum_{i=1}^{N/l_{b}}{\bar{g}}_{i}{\bar{g}}_{i}^{T}. \end{array}$$ (17)

If *N* is not an integer multiple of *l*_b_ the number of blocks is [*N*/*l*_b_], the largest integer smaller or equal *N*/*l*_b_. Then **V** is multiplied by *N*/([*N*/*l*_b_]*l*_b_) to get the final estimate.

An ensemble of size *M* of parameter vectors is generated as ${\left\{\hat{\alpha }+Lv_{i}\right\}}_{i=1}^{M}$ where **v**_*i*_ are column vectors of length *J* of independent standard normal random variables and **L** is obtained from the Cholesky decomposition **C** = **LL**^T^. Only samples satisfying the slope inequality constraints (see Subsections 4.1 and 5.1), if any, are accepted. This yields an ensemble of probability densities consistent with the parameter uncertainty which can be used to construct confidence intervals for quantities of interest, for example, quantiles of the distribution.

An alternative method for assessing parameter uncertainty is to apply bootstrap resampling, or block bootstrap resampling for dependent data, on the entire model estimation. This approach guarantees that any resampled model satisfies the inequality constraints, if any. If the exponential family is not steep it is possible that the maximum likelihood estimator does not exist for some resamples but this should not really be a problem in practice as it happens only if the model is close to the solution boundary which is an indication that it is not appropriate anyway for the data under consideration. Moreover, almost all relevant models here are steep. Bootstrap resampling is considerably more computationally costly than the method above as the optimisation has to be repeated *M* times but it is still feasible in many applications.

### 2.4 Convergence properties of the estimator

An alternative characterisation of the maximum likelihood probability density estimate $\hat{p}\left(y\right)$ is that it minimises the relative entropy or Kullback–Leibler divergence of the true probability density *f*(*y*) with respect to *p*(*y*),

$$\begin{array}{c} I\left(f|p\right)=\int_{D}f\left(y\right)\;\text{log}\frac{f(y)}{p(y)}\;dy, \end{array}$$ (18)

within the chosen exponential family [22, 23]. Note that *I*(*f*_*X*_|*p*_*X*_) = *I*(*f*|*p*). There is the Pythogorean-like decomposition

$$\begin{array}{c} I\left(f|\hat{p}\right)=I\left(f|p^{*}\right)+I\left(p^{*}|\hat{p}\right) \end{array}$$ (19)

with p* being the information projection of *f*, that is, the density from the exponential family which has the same population means of the sufficient statistics as the true density: ∫_*D*_ *ϕ*_*j*_(*y*)p*(*y*)*dy* = ∫_*D*_ *ϕ*_*j*_(*y*)*f*(*y*) *dy*; *j* = 1, …, *J*. The first term on the right hand side of Eq (19) is the approximation error due to the truncation of the exponential family; it decreases with increasing *J*. The second term is the estimation error due to the finite sample size; it decreases with increasing *N*. Convergence of the relative entropy to zero as *J* → ∞ and *N* → ∞ has been established for many practically relevant model classes such as polynomials, splines and trigonometric functions on a bounded interval [22, 23, 28] as well as under certain conditions for polynomials on infinite and semi-infinite intervals [50]. Convergence in relative entropy implies convergence in the *L*_1_-norm [51].

For bounded intervals, it has been shown [23] that if the log-density has *r* square-integrable derivatives the Kullback–Leibler divergence $I(f|\hat{p})$ converges to zero at rate 1/*J*^2*r*^ + *J*/*N* as *J* → ∞ and *J*^3^/*N* → 0 in the polynomial case and *J*^2^/*N* → 0 in the spline and trigonometric cases. By setting *J* = *N*^1/(2*r*+1)^ this specializes to the rate *N*^−2*r*/(2*r*+1)^, thus approaching the parametric rate *N*^−1^ for well-behaved log-densities. For splines this rate can only be achieved when also increasing the order of the spline with the sample size. With cubic splines it is not possible to take advantage of smoothness *r* > 4 and the convergence rate is limited to *N*^−8/9^. Convergence rates for infinite and semi-infinite intervals are harder to obtain and depend on the tail behaviour of the true density [23].

In practice, the smoothness class of the true density is not known and the dimension *J* of the exponential family should be chosen automatically from the data. When selecting the dimension by a minimum description length information criterion similar to those proposed by Schwarz [52] or Rissanen [53] the density estimator on bounded intervals converges in squared Hellinger distance at rate (*N*^−1^ log *N*)^2*r*/(2*r*+1)^ for polynomials, approaching the parametric rate *N*^−1^ up to a logarithmic factor, and at rate (*N*^−1^ log *N*)^8/9^ for cubic splines [54].

For densities which go to zero or have an integrable singularity at a boundary point convergence may be rather slow because of the singularity in the log-density. The inclusion of boundary terms in the estimator may then be very beneficial. For example, if there is power-law behaviour of the density close to a domain boundary as with, for example, the gamma distribution at zero a logarithmic boundary term absorbs this behaviour and the smoothness parameter *r* for the remainder of the log-density may increase significantly.

It should generally be kept in mind that the asymptotic behaviour of density estimators does not necessarily carry over to finite sample sizes [2]. The practical performance can only be assessed and compared through extensive numerical experiments with different types of densities and realistic finite sample sizes. Moreover, the assessment of a density estimator goes beyond integrated pointwise error measures; in particular applications interest may lie, for example, in detecting the modality of a density and changes in it over time [55, 56].

### 2.5 Statistically orthogonal basis functions

There is a gauge freedom in the representation of the potential function *U*(*y*) in the linear subspace spanned by a given set of basis functions {*ϕ*_1_, …, *ϕ*_*J*_}. When transforming the basis functions with any invertible *J* × *J* matrix and at the same time transforming the vector of expansion coefficients with the inverse matrix the potential function is invariant. Moreover, the potential function is only determined up to an arbitrary additive constant.

An empirical scalar product over the data points is defined for two functions *e*_1_(*y*) and *e*_2_(*y*) as

$$\begin{array}{c} \left(e_{1},e_{2}\right)=\frac{1}{N}\sum_{n=1}^{N}e_{1}\left(y_{n}\right)e_{2}\left(y_{n}\right). \end{array}$$ (20)

It can be interpreted as a Monte Carlo approximation to the integral ∫_*D*_ *e*_1_(*y*)*e*_2_(*y*)*f*(*y*)*dy*. The corresponding norm is

$$\begin{array}{c} \left||e|\right|=\sqrt{\left(e,e\right)}. \end{array}$$ (21)

We start with a base measure exp[*β*(*y*)] and a set of basis functions {*γ*_1_, …, *γ*_*J*_} such that the set {1, *β*, *γ*_1_, …, *γ*_*J*_} is linearly independent. The sample means of *β* and {*γ*_1_, …, *γ*_*J*_} are removed and the basis functions are orthonormalised with respect to the empirical scalar product using the Gram–Schmidt procedure. We here actually use the modified Gram–Schmidt algorithm [57] which is numerically more stable. A pseudocode is as follows:

*ψ* ← *β* − 〈*β*〉

for *j* = 1: *J*

  *ϕ*_*j*_ ← *γ*_*j*_ − 〈*γ*_*j*_〉

end

for *j* = 1: *J*

  *ϕ*_*j*_ ← *ϕ*_*j*_/||*ϕ*_*j*_||

  for *k* = *j* + 1: *J*

    *ϕ*_*k*_ ← *ϕ*_*k*_ − (*ϕ*_*j*_, *ϕ*_*k*_)*ϕ*_*j*_

  end

end

The new basis functions {*ϕ*_1_, …, *ϕ*_*J*_} satisfy

$$\begin{array}{c} \langle \phi_{j}\rangle =0 \end{array}$$ (22)

and

$$\begin{array}{c} \left(\phi_{j},\phi_{k}\right)=\delta_{jk} \end{array}$$ (23)

for *j*, *k* = 1, …, *J*. We also have 〈*ψ*〉 = 0 and thus 〈*U*〉 = 0 for all ***α***. The log-likelihood function, the score function and the likelihood equations now simplify to

$$\begin{array}{c} l=-N\;\text{log}\;Z, \end{array}$$ (24)

$$\begin{array}{c} g=-Nm \end{array}$$ (25)

and

$$\begin{array}{c} m(\hat{\alpha })=0. \end{array}$$ (26)

Also Eq (16) simplifies using Eq (22). This constitutes an approximately statistically orthogonal formulation of the inference problem as the Fisher information matrix now is

$$\begin{array}{c} J=NE_{\hat{\alpha }}\;(\phi \phi^{T})\approx NI. \end{array}$$ (27)

We still use the exact Fisher information matrix to calculate the parameter error covariance matrix. The orthonormality of the basis over the data set guarantees a very low condition number of the log-likelihood maximisation close to the solution for any reasonable density model. This is an improvement on the use of classical orthogonal polynomials such as Legendre polynomials [28] or *B*-splines [29, 31] as well-conditioned bases, particularly for rather high-dimensional models. In the context of the moment-constrained maximum entropy problem the present technique generalises the ideas of constraint rescaling and orthogonalisation of the basis functions [37]. Note that the orthogonalisation is here performed only once before starting the maximisation of the likelihood as opposed to the repeated reorthogonalisation with respect to the current modelled probability density proposed in [37].

### 2.6 Possible regularisation of the log-likelihood function

We remark in passing that a regularisation term could be added to the log-likelihood function in line with the idea of maximum penalized likelihood [24–27]. One would then, for example, minimise the (convex) function

$$-l+\eta {|\alpha -\alpha_{0}|}_{2}^{2}$$

rather than maximise *l*. Here, ***α***_0_ corresponds to a reference probability density which could be uniform, normal and exponential or gamma for the bounded, infinite and semi-infinite domain, respectively. Other choices are possible if there is prior knowledge about the data supporting them. The non-negative regularisation parameter *η* could be determined by cross-validation. This regularisation is equivalent to a Bayesian approach which puts a Gaussian prior on ***α***, centred at ***α***_0_, with diagonal covariance matrix and equal variance for all components inverse proportional to *η*. This line is not pursued in the present paper as we focus on enforcing parsimony with the Bayesian information criterion without any penalty term.

## 3 Bounded interval

We first model probability densities supported on a known bounded interval *D*_*X*_ = [*a*, *b*]. This includes cases where the potential function or the density is actually defined only on [*a*, *b*), (*a*, *b*] or (*a*, *b*). The domain *D*_*X*_ is mapped onto the domain *D* = [−1, 1] by the rescaling *y* = (2*x* − *a* − *b*)/(*b* − *a*) and the data sample is transformed accordingly. The probability density *f*(*y*) is related to *f*_*X*_(*x*) by *f*((2*x* − *a* − *b*)/(*b* − *a*)) = [(*b* − *a*)/2]*f*_*X*_(*x*).

We have

$$\begin{array}{c} Z=\int_{-1}^{1}\text{exp}\left[U\left(y\right)\right]\;dy, \end{array}$$ (28)

$$\begin{array}{c} m_{j}=\int_{-1}^{1}\phi_{j}\left(y\right)\text{exp}\left[V\left(y\right)\right]\;dy, \end{array}$$ (29)

and

$$\begin{array}{c} Q_{jk}=\int_{-1}^{1}\phi_{j}\left(y\right)\phi_{k}\left(y\right)\text{exp}\left[V\left(y\right)\right]\;dy. \end{array}$$ (30)

The integrals are evaluated using Gauss–Legendre quadrature.

### 3.1 Polynomial basis functions

The basis functions are *γ*_*j*_(*y*) = *y*^*j*^ or more generally $\gamma_{j}\left(y\right)=y^{k_{j}}$ with distinct positive integers *k*_*j*_ if there is prior knowledge to justify the use of particular powers. There is no point here in using any orthogonal polynomials as a statistically orthogonal basis is constructed.

If the density *f*(*y*) is known to be symmetric about zero only even powers would be used. If the density is periodic, for example, a density over angles (*D*_*X*_ = [0, 2*π*]), the constraint *U*(−1) = *U*(1) would be imposed. There are not necessarily any constraints on the derivatives of *U*. Given a polynomial basis ${\left\{\gamma_{j}^{*}\right\}}_{j=1}^{J+1}$ a basis ${\left\{\gamma_{j}\right\}}_{j=1}^{J}$ satisfying *γ*_*j*_(−1) = *γ*_*j*_(1) can be constructed from any basis of the null space of the 1 × (*J* + 1) constraint matrix

$$(\begin{array}{ccc} \gamma_{1}^{*}\left(-1\right)-\gamma_{1}^{*}\left(1\right) & \cdots & \gamma_{J+1}^{*}\left(-1\right)-\gamma_{J+1}^{*}\left(1\right) \end{array}),$$

spanned by the right singular vectors corresponding to the zero singular values.

### 3.2 Spline basis functions

The construction of splines on the interval [*y*_l_, *y*_u_] is described. Here, the case *y*_l_ = −1 and *y*_u_ = 1 is relevant.

Given a sequence of (simple) knots *y*_l_ < *ζ*_1_ < … < *ζ*_*K*_ < *y*_u_ with *K* ≥ 1 a spline function is a twice continuously differentiable function whose restriction on each of the *K* + 1 sections [*y*_l_, *ζ*_1_], [*ζ*_1_, *ζ*_2_], …, [*ζ*_*K*−1_, *ζ*_*K*_], [*ζ*_*K*_, *y*_u_] is a cubic polynomial. A spline function can be represented as

$$\begin{array}{c} \gamma \left(y\right)=\sum_{k=1}^{K+1}\sum_{i=0}^{3}\beta_{ik}\xi_{ik}\left(y\right) \end{array}$$ (31)

with $\xi_{ik}\left(y\right)={\left(y-{\bar{y}}_{k}\right)}^{i}$ on the *k*th section and *ξ*_*ik*_(*y*) = 0 otherwise. Here, ${\bar{y}}_{k}$ is the midpoint of the *k*th section. The expansion coefficients {*β*_*ik*_} are constrained by the requirement of continuity of *γ*, *γ*′ and *γ*″ at the knots. It is convenient to also immediately include here the constraint 〈*γ*〉 = ∑_*k*_ ∑_*i*_ *β*_*ik*_〈*ξ*_*ik*_〉 = 0 as a constant function on *D* = [−1, 1] is still contained in the space spanned by the functions {*ξ*_*ik*_}. These are 3*K* + 1 homogeneous linear constraints on the 4*K* + 4 expansion coefficients {*β*_*ik*_} which are arranged in a sparse (3*K* + 1) × (4*K* + 4) matrix. The spline functions form a linear space of dimension *K* + 3 spanned by a linearly independent set ${\left\{\gamma_{j}\right\}}_{j=1}^{K+3}$. A basis can be constructed from any basis of the null space of the constraint matrix, spanned by the right singular vectors corresponding to the zero singular values. A more localised basis leading to a sparser Hessian matrix **H** could be obtained by referring to *B*-splines [31] but sparsity will be lost anyway when going to the statistically orthogonal basis functions and the overall computation time of the density estimation is not significantly affected by this. Note that the splines here are not natural splines; there are no constraints at the end points *y* = *y*_l_ and *y* = *y*_u_ which are not knots and the curvature there may be different from zero. The model is more flexible this way and there is no reason for a zero curvature constraint in the context of density estimation.

For the position of the knots we study two canonical choices: equally spaced knots in *y*-space,

$$\begin{array}{c} \zeta_{k}=y_{l}+\frac{k}{K+1}\left(y_{u}-y_{l}\right),\;k=1,\ldots ,K, \end{array}$$ (32)

and equally spaced knots with respect to the empirical distribution function,

$$\begin{array}{c} \zeta_{k}=y_{n_{k}}^{*},\;k=1,\ldots ,K, \end{array}$$ (33)

where $\left\{y_{1}^{*},\ldots ,y_{N}^{*}\right\}$ is the data sample sorted into non-decreasing order and *n*_*k*_ is the largest integer smaller or equal to *kN*/(*K* + 1).

For symmetric probability densities the knots are chosen symmetric and the symmetry conditions are added to the constraint matrix. The symmetry conditions are not all independent from the knot conditions and the singular value decomposition is used to determine the rank of the constraint matrix and thus the dimension of the spline space and a basis of it. If the density is periodic the condition *γ*(*y*_l_) = *γ*(*y*_u_) is added to the constraint matrix.

### 3.3 Trigonometric basis functions

A linearly independent set of basis functions of size *J* = 2*K* is ${\left\{\text{cos}(k\pi /2)y,\sin(k\pi /2)y\right\}}_{k=1}^{K}$. For a symmetric probability density an appropriate set is ${\left\{\text{cos}(j\pi /2)y\right\}}_{j=1}^{J}$. If the density is periodic a basis can be constructed analogously to the polynomial case.

### 3.4 Boundary basis functions

For probability densities with a zero value or an integrable singularity at any of the boundary points *y* = −1 and *y* = 1 the model can be considerably improved by including an appropriate subset of the boundary basis functions {log(*y* + 1), log(1 − *y*), 1/(*y* + 1), 1/(1 − *y*)}. In practice, the logarithmic terms are most often used to model power-law decay of the density to zero near a boundary point as, for example, in the beta distribution. In case of symmetry the boundary terms would be introduced simultaneously at both boundaries.

## 4 Infinite interval

We now look at probability densities on the infinite interval *D*_*X*_ = (−∞, ∞). The domain *D*_*X*_ is mapped onto itself with the rescaling *y* = (*x* − *μ*)/*σ* where *μ* is the sample mean and *σ* is the sample standard deviation. The probability density *f*(*y*) is linked to *f*_*X*_(*x*) by *f*((*x* − *μ*)/*σ*) = *σf*_*X*_(*x*). If *f*_*X*_(*x*) is known to be symmetric about x_*_ the rescaling *y* = (*x*−x_*_)/*σ* is applied with *σ* being the standard deviation about x_*_.

### 4.1 Linearly extrapolated basis functions

Given a set of basis functions ${\left\{\phi_{0,j}\right\}}_{j=1}^{J}$ we introduce linearly extrapolated basis functions as

$$\begin{array}{c} \phi_{j}\left(y\right)=\{\begin{array}{cc} \phi_{0,j}\left(y\right) & y_{a}\leq y\leq y_{b}\\ \phi_{0,j}\left(y_{b}\right)+\phi_{0,j}^{\prime }\left(y_{b}\right)\left(y-y_{b}\right) & y_{b}<y\\ \phi_{0,j}\left(y_{a}\right)+\phi_{0,j}^{\prime }\left(y_{a}\right)\left(y-y_{a}\right) & y<y_{a} \end{array} \end{array}$$ (34)

where *y*_a_ and *y*_b_ are the smallest and largest, respectively, data point in the data sample. We still have *U*(*y*) = ∑_*j*_ *α*_*j*_ *ϕ*_*j*_(*y*) for all *y* and impose the slope conditions

$$\begin{array}{c} U^{\prime }\left(y_{a}\right)=\sum_{j}\alpha_{j}\phi_{j}^{\prime }\left(y_{a}\right)>0 \end{array}$$ (35)

and

$$\begin{array}{c} U^{\prime }\left(y_{b}\right)=\sum_{j}\alpha_{j}\phi_{j}^{\prime }\left(y_{b}\right)<0 \end{array}$$ (36)

in order to have a proper probability density. The functions ${\left\{\phi_{0,j}\right\}}_{j=1}^{J}$ may be polynomials or splines. Trigonometric functions are probably less efficient here as the density usually strongly decays at the points *y* = *y*_a_ and *y* = *y*_b_ given the infinite domain of support.

We do not aim at any rigorous extreme value modelling here. By construction there are no data points in the exponential tails and the tail model is just the lowest order extrapolation of the bulk model. The probability mass accounted for by the tails is of order 1/*N*. The probability density has only one continuous derivative at *y* = *y*_a_ and *y* = *y*_b_. Thus the coupling of the bulk model to the tail is rather weak and so is also the influence of the particular choice of tail model on the overall model.

The partition function is

$$\begin{array}{c} Z=Z_{0}+Z_{1}+Z_{2} \end{array}$$ (37)

with

$$\begin{array}{ccc} Z_{0} & = & \int_{y_{a}}^{y_{b}}\text{exp}\left[U\left(y\right)\right]\;dy \end{array}$$ (38)

$$\begin{array}{ccc} Z_{1} & = & \int_{y_{b}}^{\infty }\text{exp}\left[U\left(y\right)\right]\;dy=-\frac{\text{exp}[U\left(y_{b}\right)]}{U^{\prime }\left(y_{b}\right)} \end{array}$$ (39)

$$\begin{array}{ccc} Z_{2} & = & \int_{-\infty }^{y_{a}}\text{exp}\left[U\left(y\right)\right]\;dy=\frac{\text{exp}[U\left(y_{a}\right)]}{U^{\prime }\left(y_{a}\right)}. \end{array}$$ (40)

We have

$$\begin{array}{c} m_{j}=m_{0,j}+m_{1,j}+m_{2,j} \end{array}$$ (41)

with

$$\begin{array}{ccc} m_{0,j} & = & \int_{y_{a}}^{y_{b}}\phi_{j}\left(y\right)\text{exp}\left[V\left(y\right)\right]\;dy \end{array}$$ (42)

$$\begin{array}{ccc} m_{1,j} & = & \int_{y_{b}}^{\infty }\phi_{j}\left(y\right)\text{exp}\left[V\left(y\right)\right]\;dy=\frac{Z_{1}}{Z}[\phi_{j}\left(y_{b}\right)-\frac{\phi_{j}^{\prime }\left(y_{b}\right)}{U^{\prime }\left(y_{b}\right)}] \end{array}$$ (43)

$$\begin{array}{ccc} m_{2,j} & = & \int_{-\infty }^{y_{a}}\phi_{j}\left(y\right)\text{exp}\left[V\left(y\right)\right]\;dy=\frac{Z_{2}}{Z}[\phi_{j}\left(y_{a}\right)-\frac{\phi_{j}^{\prime }\left(y_{a}\right)}{U^{\prime }\left(y_{a}\right)}] \end{array}$$ (44)

and

$$\begin{array}{c} Q_{jk}=Q_{0,jk}+Q_{1,jk}+Q_{2,jk} \end{array}$$ (45)

with

$$\begin{array}{ccc} Q_{0,jk} & = & \int_{y_{a}}^{y_{b}}\phi_{j}\left(y\right)\phi_{k}\left(y\right)\text{exp}\left[V\left(y\right)\right]\;dy \end{array}$$ (46)

$$\begin{array}{ccc} Q_{1,jk} & = & \int_{y_{b}}^{\infty }\phi_{j}\left(y\right)\phi_{k}\left(y\right)\text{exp}\left[V\left(y\right)\right]\;dy \end{array}$$ (47)

$$\begin{array}{ccc} & = & \frac{Z_{1}}{Z}\{[\phi_{j}\left(y_{b}\right)-\frac{\phi_{j}^{\prime }\left(y_{b}\right)}{U^{\prime }\left(y_{b}\right)}][\phi_{k}\left(y_{b}\right)-\frac{\phi_{k}^{\prime }\left(y_{b}\right)}{U^{\prime }\left(y_{b}\right)}]+\frac{\phi_{j}^{\prime }\left(y_{b}\right)\phi_{k}^{\prime }\left(y_{b}\right)}{{\left[U^{\prime }\left(y_{b}\right)\right]}^{2}}\} \end{array}$$ (48)

$$\begin{array}{ccc} Q_{2,jk} & = & \int_{-\infty }^{y_{a}}\phi_{j}\left(y\right)\phi_{k}\left(y\right)\text{exp}\left[V\left(y\right)\right]\;dy \end{array}$$ (49)

$$\begin{array}{ccc} & = & \frac{Z_{2}}{Z}\{[\phi_{j}\left(y_{a}\right)-\frac{\phi_{j}^{\prime }\left(y_{a}\right)}{U^{\prime }\left(y_{a}\right)}][\phi_{k}\left(y_{a}\right)-\frac{\phi_{k}^{\prime }\left(y_{a}\right)}{U^{\prime }\left(y_{a}\right)}]+\frac{\phi_{j}^{\prime }\left(y_{a}\right)\phi_{k}^{\prime }\left(y_{a}\right)}{{\left[U^{\prime }\left(y_{a}\right)\right]}^{2}}\}. \end{array}$$ (50)

The integrals in Eqs (38), (42) and (46) are calculated by Gauss–Legendre quadrature.

Linearly extrapolated polynomials as basis functions have a couple of advantages over the standard polynomials as the topology of the parameter space is more favourable:

(i) Linearly extrapolated polynomials generate regular exponential families as opposed to standard polynomials generating models which are generally not regular and not steep (see S1 Appendix). The maximum likelihood estimator is guaranteed to exist for any generic data sample. As a simple example for lack of steepness, the model *U*(*y*) = *α*_1_ *y*^2^ + *α*_2_ *y*^4^ with standard polynomials has the upper bound $\mu_{4}\leq 3\mu_{2}^{2}$ on the moments (see Theorem 3 in S2 Appendix). This upper boundary is formed by the Gaussian distributions corresponding to *α*_1_ < 0 and *α*_2_ = 0. The model cannot cover any leptocurtic distributions. With linearly extrapolated polynomials there is no such restriction as the model can use a positive coefficient *α*_2_ as long as the slope conditions $2\alpha_{1}y_{a}+4\alpha_{2}y_{a}^{3}>0$ and $2\alpha_{1}y_{b}+4\alpha_{2}y_{b}^{3}<0$ are met.
(ii) The standard polynomials require the highest power in the model to be even in order to get a proper probability density. There is no restriction with linearly extrapolated polynomials. The number of parameters can be increased in steps of one which may lead to more parsimonious models.
(iii) The numerical evaluation of the integrals involved in the log-likelihood function and its derivatives is more stable for the linearly extrapolated polynomials as the tail parts are treated analytically. With standard polynomials one would use Gauss–Hermite quadrature which may become inaccurate and unreliable close to the boundary of the parameter space, that is, if the leading-order coefficient is close to zero. As a consequence the numerical maximization of the likelihood function might not converge to the solution even if it still exists.
(iv) With standard polynomials solutions close to the boundary of the parameter space occur naturally. When doing the model selection one would gradually increase the number of basis functions. At some point, additional terms are not needed any more which manifests itself in the leading-order coefficient being close to zero. With linearly extrapolated polynomials typical solutions are located away from the boundary of the parameter space as on the infinite domain probability densities are usually clearly decaying at both extreme data points *y* = *y*_a_ and *y* = *y*_b_. Solutions with one or both of the slopes *U*′(*y*_a_) and *U*′(*y*_b_) close to zero are possible, particularly for small and slightly atypical data samples, but they are very unlikely.

In the spline case, a spline basis ${\left\{\phi_{j}\right\}}_{j=1}^{K+3}$ on the interval [*y*_a_, *y*_b_] is constructed as described in Subsection 3.2 and then linearly extrapolated as described above. We do not use natural splines as opposed to [31]. A zero curvature condition at the extremal points *y* = *y*_a_ and *y* = *y*_b_ appears to be artificial or even detrimental to the model. It is only justified if the density actually has an exponential tail. The present setting is more flexible; the extremal points are not knots and the density has generally only one continuous derivative there as opposed to two at the knots. We also do not use any preliminary tail transformation here.

For a symmetric probability density a linearly extrapolated polynomial or spline basis is set up as before on the symmetric interval [-max(|*y*_a_|, |*y*_b_|), max(|*y*_a_|, |*y*_b_|)].

## 5 Semi-infinite interval

Finally, we model probability densities supported on the semi-infinite interval *D*_*X*_ = [*a*, ∞) with known lower bound *a*. This includes cases where the potential function or the density is actually defined only on (*a*, ∞). The case *D*_*X*_ = (−∞, *b*] is readily referred back to the case *D*_*X*_ = [*a*, ∞) with *a* = −*b* by considering the variable −*X* instead of *X*. The interval *D*_*X*_ is mapped onto the interval *D* = [0, ∞) with the rescaling *y* = (*x* − *a*)/*μ* where *μ* is the sample mean of *X* − *a*. The probability density *f*(*y*) is linked to *f*_*X*_(*x*) by *f*((*x* − *a*)/*μ*) = *μf*_*X*_(*x*).

### 5.1 Linearly extrapolated basis functions

Again, linearly extrapolated basis functions are introduced as

$$\begin{array}{c} \phi_{j}\left(y\right)=\{\begin{array}{cc} \phi_{0,j}\left(y\right) & 0\leq y\leq y_{b}\\ \phi_{0,j}\left(y_{b}\right)+\phi_{0,j}^{\prime }\left(y_{b}\right)\left(y-y_{b}\right) & y_{b}<y \end{array} \end{array}$$ (51)

where *y*_b_ is the largest data point in the data sample. The slope condition

$$\begin{array}{c} U^{\prime }\left(y_{b}\right)=\sum_{j}\alpha_{j}\phi_{j}^{\prime }\left(y_{b}\right)<0 \end{array}$$ (52)

guarantees a normalisable probability density. The functions ${\left\{\phi_{0,j}\right\}}_{j=1}^{J}$ are polynomials or splines, now constructed on the interval [0, *y*_b_].

The partition function is

$$\begin{array}{c} Z=Z_{0}+Z_{1} \end{array}$$ (53)

with

$$\begin{array}{c} Z_{0}=\int_{0}^{y_{b}}\text{exp}\left[U\left(y\right)\right]\;dy \end{array}$$ (54)

and *Z*_1_ given by Eq (39). We have

$$\begin{array}{c} m_{j}=m_{0,j}+m_{1,j} \end{array}$$ (55)

with

$$\begin{array}{c} m_{0,j}=\int_{0}^{y_{b}}\phi_{j}\left(y\right)\text{exp}\left[V\left(y\right)\right]\;dy \end{array}$$ (56)

and *m*_1,*j*_ as in Eq (43) as well as

$$\begin{array}{c} Q_{jk}=Q_{0,jk}+Q_{1,jk} \end{array}$$ (57)

with

$$\begin{array}{c} Q_{0,jk}=\int_{0}^{y_{b}}\phi_{j}\left(y\right)\phi_{k}\left(y\right)\text{exp}\left[V\left(y\right)\right]\;dy \end{array}$$ (58)

and *Q*_1,*jk*_ as in Eq (48). The integrals in Eqs (54), (56) and (58) are again evaluated using Gauss–Legendre quadrature.

The advantages of linearly extrapolated polynomials over standard polynomials carry over accordingly from the infinite to the semi-infinite domain. A simple example for lack of steepness is the model *U*(*y*) = *α*_1_ *y* + *α*_2_ *y*^2^, the truncated normal distribution, which has the upper bound $\mu_{2}\leq 2\mu_{1}^{2}$ on the moments (see Theorem 3 in S2 Appendix). The boundary is formed by the exponential distributions corresponding to *α*_1_ < 0 and *α*_2_ = 0. The model can only cover distributions with a coefficient of variation less than or equal to 1. The corresponding linearly truncated model is regular and has no such restriction as it can use a positive coefficient *α*_2_ as long as the slope condition *α*_1_ + 2*α*_2_ *y*_b_ < 0 is met.

### 5.2 Boundary basis functions

The basis may be augmented (prior to orthonormalisation) with an appropriate subset of the functions {log *y*, 1/*y*, log^2^ *y*}, all linearly extrapolated at *y*_b_. We do not include the function log^2^ *y* without also including log *y* as then the invariance of the exponential family under linear rescaling (see Subsection 2.1) would be violated. The functions log*y* and 1/*y* are genuine boundary terms modelling decay to zero or an integrable singularity of the density at *y* = 0 whereas the function log^2^ *y* may be useful for densities close to a log-normal distribution. In practice, most often just the boundary term log *y* is used to model power-law decay to zero at *y* = 0 as, for example, in the gamma distribution.

## 6 Maximising the likelihood function

The log-likelihood function is iteratively maximised with respect to the parameters ***α*** using a modified Newton–Raphson method which combines the rapid quadratic convergence of the Newton–Raphson method close to the solution with a guarantee of global convergence by ensuring an increase of the likelihood function at each iteration. Having arrived after *k* iterations at an estimate ***α***^(*k*)^ with gradient vector **g**^(*k*)^ and Hessian matrix **H**^(*k*)^ the search direction **p**^(*k*+1)^ for the next iteration is given by

$$\begin{array}{c} (M^{\left(k\right)}+\eta^{\left(k+1\right)}I)p^{\left(k+1\right)}=-m^{\left(k\right)} \end{array}$$ (59)

with **M** = −*N*^−1^ **H** where *η* ≥ 0 is a regularisation parameter. The vector **p**^(*k*+1)^ is an ascent direction of the likelihood function for any choice of *η*^(*k*+1)^; we have (dropping the iteration superscripts for convenience)

$$\begin{array}{c} N^{-1}p^{T}g=m^{T}{\left(M+\eta I\right)}^{-1}m>0 \end{array}$$ (60)

for **m** ≠ 0 as **M** is positive definite. An inexact line search along the regularised Newton–Raphson direction **p**^(*k*+1)^ is performed using a simple backtracking of the step size. The parameter vector is updated from ***α***^(*k*)^ to

$$\begin{array}{c} \alpha^{\left(k+1\right)}=\alpha^{\left(k\right)}+\omega^{\left(k+1\right)}p^{\left(k+1\right)} \end{array}$$ (61)

with *ω*^(*k*+1)^ = 2^−*i*^ where *i* is the smallest non-negative integer such that *l*(***α***^(*k*+1)^) > *l*(***α***^(*k*)^) and ***α***^(*k*+1)^ satisfies the slope condition(s) (see Subsections 4.1 and 5.1), if any.

The regularisation parameter *η* is introduced to ward off possible ill-conditioning of the Hessian, particularly in the early iterations of the maximisation when the modelled probability density is still far away from the true probability density. It is here chosen automatically such that the condition number of the regularised Hessian does not exceed a prescribed threshold *κ*_th_. The spectral condition number of the Hessian is equal to the Rayleigh ratio *κ* = λ_max_/λ_min_ where λ_max_ and λ_min_ are the largest and smallest eigenvalues, respectively, of **M**. If *κ* ≤ *κ*_th_ then *η* = 0; otherwise *η* = (λ_max_ − *κ*_th_λ_min_)/(*κ*_th_ − 1) ≈ λ_max_/*κ*_th_ − *λ*_min_. We set *κ*_th_ = 10^5^; the choice of *κ*_th_ influences only the optimisation path but not the solution. Spline models tend to have better condition than polynomial models. Due to the approximate statistical orthonormality of the basis functions (see Subsection 2.5) the inference is well-conditioned close to the solution for any reasonable parsimonious model.

The iteration is started with an initial guess parameter vector ***α***^(0)^ satisfying the slope condition(s) (see Subsections 4.1 and 5.1), if any. For a bounded interval a simple and robust choice is ***α***^(0)^ = 0. For the infinite interval, ***α***^(0)^ is chosen to minimise $\int_{y_{a}}^{y_{b}}{\left[U(y)\right]}^{2}\;dy$ subject to *U*′(*y*_a_) = 1 and *U*′(*y*_b_) = −1; for a semi-infinite interval ***α***^(0)^ is chosen to minimise $\int_{0}^{y_{b}}{\left[U(y)\right]}^{2}\;dy$ subject to *U*′(*y*_b_) = −1. These are readily found as solutions to standard least-squares problems. For nested models of increasing order, for example, polynomial or trigonometric, the iteration can be started with the solution of the previous model.

The iteration is terminated as soon as |**m**^(*k*)^| < *ε* with *ε* = 10^−5^. Only models satisfying this criterion within a prescribed maximum number of iterations are included in the model selection described in Section 7. Failure to reach a solution may occur if the exponential family is not steep and the maximum likelihood estimator does not exist (see S1 Appendix), if the solution lies extremely close to the boundary of the parameter space or if the problem is very ill-conditioned. All these reasons indicate that the model is not adequate anyway for the particular data. We here set the maximum number of iterations to 50; adequate models are usually found with fewer than 15 iterations.

The calculation of the log-likelihood, the gradient and the Hessian does not require any reference to the data sample and thus the computation time does not significantly increase with the sample size *N* for common sample sizes. It increases linearly with the number of quadrature nodes used in the numerical integrations which is usually much smaller than the sample size. Only for very large sample sizes the initial orthonormalisation becomes dominant and the algorithm scales linearly with *N*.

## 7 Model selection

Table 1 summarises the possible configurations of basis functions for the different domains. Model selection is performed with the Bayesian information criterion (BIC) [52]:

$$\begin{array}{c} \text{BIC}=J\;\text{log}\;N-2l(\hat{\alpha }) \end{array}$$ (62)

The number of basis functions in the exponential family is *J* = *J*_b_ + *J*_0_ where *J*_b_ is the number of boundary terms and *J*_0_ is the number of bulk basis functions. The BIC is minimised over both the number and type of basis functions taking into account models up to a prescribed maximum number of basis functions, reflecting the maximum complexity of the density estimate. The BIC has been shown to pick the correct exponential family with probability tending to 1 as *N* → ∞ if the true density is in one of the exponential families [58]. Usually, the complexity of the chosen model increases with increasing sample size *N*.

**Table 1:** Overview of domains and basis functions for semiparametric density estimation.

Domain	Rescaling	Basis functions {*γ*_1_, …, *γ*_*J*_}
*D*_*X*_ = [*a*, *b*] *D* = [−1, 1]	$y=\frac{2x-a-b}{b-a}$	$\left\{\text{log}\left(y+1\right),\text{log}\left(1-y\right),\frac{1}{y+1},\frac{1}{1-y}\right\}$ + {*y*, *y*^2^, …}
		$\left\{\text{log}\left(y+1\right),\text{log}\left(1-y\right),\frac{1}{y+1},\frac{1}{1-y}\right\}$ + splines
		$\left\{\text{log}\left(y+1\right),\text{log}\left(1-y\right),\frac{1}{y+1},\frac{1}{1-y}\right\}$ + {cos(*kπ*/2)*y*, sin(*kπ*/2)*y*, cos *kπy*, sin *kπy*, …}
*D*_*X*_ = [*a*, *b*] symmetric about $\frac{a+b}{2}$ *D* = [−1, 1] symmetric about 0	$y=\frac{2x-a-b}{b-a}$	$\left\{\text{log}\left(y+1\right)+\text{log}\left(1-y\right),\frac{1}{y+1}+\frac{1}{1-y}\right\}$ + {*y*^2^, *y*^4^, …}
		$\left\{\text{log}\left(y+1\right)+\text{log}\left(1-y\right),\frac{1}{y+1}+\frac{1}{1-y}\right\}$ + even splines
		$\left\{\text{log}\left(y+1\right)+\text{log}\left(1-y\right),\frac{1}{y+1}+\frac{1}{1-y}\right\}$ + {cos(*kπ*/2)*y*, cos *kπy*, …}
*D*_*X*_ = (−∞, ∞) *D* = (−∞, ∞)	$y=\frac{x-\mu }{\sigma }$ $\mu =\frac{1}{N}\sum_{n}x_{n}$ $\sigma =\frac{1}{N}\sum_{n}{\left(x_{n}-\mu \right)}^{2}$	{*y*, *y*^2^, …, } on [*y*_a_, *y*_b_] + linear extrapolation
		splines on [*y*_a_, *y*_b_] + linear extrapolation
*D*_*X*_ = (−∞, ∞) symmetric about x_*_ *D* = (−∞, ∞) symmetric about 0	$y=\frac{x-x_{*}}{\sigma }$ $\sigma =\frac{1}{N}\sum_{n}{\left(x_{n}-x_{*}\right)}^{2}$	{*y*^2^, *y*^4^, …, } on [*y*_a_, *y*_b_] + linear extrapolation
		even splines on [*y*_a_, *y*_b_] + linear extrapolation
*D*_*X*_ = [*a*, ∞) *D* = [0, ∞)	$y=\frac{x-a}{\mu }$ $\mu =\frac{1}{N}\sum_{n}\left(x_{n}-a\right)$	$\left\{\text{log}y,\frac{1}{y},{\text{log}}^{2}y\right\}$ on [0, *y*_b_] + {*y*, *y*_2_, …} on [0, *y*_b_] + linear extrapolation
		$\left\{\text{log}y,\frac{1}{y},{\text{log}}^{2}y\right\}$ on [0, *y*_b_] + splines on [0, *y*_b_] + linear extrapolation

### 7.1 Spline knot deletion scheme

The spline models are refined with a knot deletion strategy in two steps:

(i) Firstly, always only models with at least 4 data points in any section are considered regardless of the BIC. Going from left to right if a section has less than 4 data points the left knot is removed and the procedure repeated until each section has at least 4 data points. It is possible, though highly unlikely, that all knots are removed; then the model is dropped. Note that this first knot deletion occurs only for equally spaced knots (Eq (32)); for knots placed according to the empirical distribution (Eq (33)), there are by construction always more than 4 data points in any section for any reasonable number of knots.
(ii) Secondly, a simple greedy knot deletion algorithm is applied: We consider sequences of knots with the number of knots increasing from *K* = 1 to a maximum number of knots *K* = *K*_m_, placed according to one of the two knot placement schemes introduced in Subsection 3.2. For each sequence, the knot deletion scheme (i) is applied and the maximum likelihood estimator is found, if any. The minimum BIC model out of these at most *K*_m_ models is picked. Each of the knots in the model is removed in turn and the reduced-order model with the lowest BIC is kept. This is repeated until removal of any of the knots does not provide a decrease of the BIC any more. As opposed to [31] we do not apply a minimum number of knots as a function of sample size to start the knot deletion from. There is no convincing reason for this and it appears to be often very likely to find more parsimonious models when increasing the number of knots starting from *K* = 1.

### 7.2 The semiparametric density estimator (SPDE)

The proposed semiparametric density estimation algorithm is as follows. This refers to general densities without symmetries or periodic behaviour; otherwise obvious modifications apply.

- Polynomials (POL):
  For bounded and semi-infinite intervals, choose the subsets of boundary terms to consider, if any. Choose the maximum polynomial order *P*_m_. For *J*_0_ = 0, 1, …, *P*_m_ (bounded interval), *J*_0_ = 2, 3, …, *P*_m_ (infinite interval) or *J*_0_ = 1, 2, …, *P*_m_ (semi-infinite interval) find the maximum likelihood estimator, separately for each subset of boundary terms. The minimum BIC model out of all these models is the polynomial density estimator POL.
- Splines with equidistant knots (SPL1):
  For bounded and semi-infinite intervals, choose the subsets of boundary terms to consider, if any. Choose the maximum number of knots *K*_m_, corresponding to a maximum number of bulk basis functions of *K*_m_ + 3. Separately for each subset of boundary terms, do the spline model selection with knot deletion as described above, placing the knots as given in Eq (32). The minimum BIC model over the subsets of boundary terms is the density estimator SPL1.
- Splines with equidistant knots with respect to the empirical distribution (SPL2):
  For bounded and semi-infinite intervals, choose the subsets of boundary terms to consider, if any. Choose the maximum number of knots *K*_m_, corresponding to a maximum number of bulk basis functions of *K*_m_ + 3. Separately for each subset of boundary terms, do the spline model selection with knot deletion as described above, placing the knots as given in Eq (33). The minimum BIC model over the subsets of boundary terms is the density estimator SPL2.
- Trigonometric functions (TRIG), only on bounded intervals:
  Choose the subsets of boundary terms to consider, if any. Choose the maximum wavenumber *L*_m_, corresponding to a maximum number of bulk basis functions of 2*L*_m_. For *J*_0_ = 0, 1, …, 2*L*_m_ find the maximum likelihood estimator, separately for each subset of boundary terms. The minimum BIC model out of all these models is the trigonometric density estimator TRIG.
- Semiparametric density estimator (SPDE):
  The semiparametric density estimator, referred to as SPDE, is the minimum BIC model out of POL, SPL1, SPL2 and TRIG.

The hyperparameters *P*_m_, *K*_m_ and *L*_m_ can usually be chosen generously high such that no model with a lower BIC is found any more when increasing them further. The choice of subsets of boundary terms is slightly subjective and motivated by visual inspection of and prior knowledge about the data. We anticipate that most often the logarithmic terms will be used to model power-law behaviour near a boundary point. Usually, the subsets of boundary terms considered would be the same for all types of bulk basis functions.

We remark in passing that a more extensive model selection would be conceivable involving arbitrary subsets of the bulk basis functions rather than just adding them one by one. If the true density is not too complex this would still be feasible in many applications but we do not discuss this here for simplicity.

## 8 Results

The semiparametric density estimation approach is applied to a range of simulation and observation data sets and compared to established alternative methodologies. These are the Gaussian kernel density estimator with three different bandwidth selection rules [1–3, 7], two variants of the diffusion estimator [7], finite mixture models [15] and three variants of local likelihood density estimation [16, 17]. An overview of the density estimation techniques is given in Table 2 and all of the details are described in S3 Appendix.

**Table 2:** Overview of density estimators. See S3 Appendix for details.

Acronym	Description
KDE1	Gaussian kernel density estimator with rule-of-thumb bandwidth [1];
	reflection method applied at any boundary point
KDE2	MATLAB in-built Gaussian kernel density estimator with default bandwidth; reflection method applied at any boundary point
KDE3	Gaussian kernel density estimator with plug-in bandwidth proposed in [7]; improved Sheather–Jones method; free from normal reference rule; reflection method applied at any boundary point
DE0	Diffusion estimator with *ρ* = 0 [7]; similar to Gaussian kernel density estimator with variable bandwidth [12]
DE1	Diffusion estimator with *ρ* = 1 [7]; similar to data sharpening method [13]
BMM	Beta mixture model [15]; number of components selected by BIC
GMM	Gaussian mixture model [15]; number of components selected by BIC
GaMM	Gamma mixture model [15]; number of components selected by BIC
WMM	Weibull mixture model [15]; number of components selected by BIC
POL	Exponential families generated by (linearly extrapolated) polynomials; optional boundary terms; model selection by BIC
SPL1	Exponential families generated by (linearly extrapolated) splines; equidistant knots; knot deletion; optional boundary terms; model selection by BIC
SPL2	Exponential families generated by (linearly extrapolated) splines; equidistant knots with respect to the empirical distribution; knot deletion; optional boundary terms; model selection by BIC
TRIG	Exponential families generated by trigonometric functions; optional boundary terms; model selection by BIC
SPDE	Best model out of POL, SPL1, SPL2 and TRIG selected by BIC
LLDE0	Local likelihood density estimator [16, 17] with constant model
LLDE1	Local likelihood density estimator [16, 17] with linear model
LLDE2	Local likelihood density estimator [16, 17] with quadratic model

The Gaussian kernel density estimators include a bandwidth selection technique proposed relatively recently by Botev et al. [7] which is an improvement on the classical Sheather–Jones solve-the-equation rule [5]. It is free from the normal reference rule and shows superior practical performance compared to existing plug-in implementations [7].

For bounded and semi-infinite domains, all of the Gaussian kernel density estimators are always combined with the reflection method applied at any boundary point as a standard boundary correction scheme. We here do not compare against more sophisticated boundary correction schemes (for example, [11]) as we compare against the diffusion estimator which has been shown to be superior to common boundary correction schemes [7]. The diffusion estimator is an interesting competitor, particularly for domains with boundaries, as it handles any boundary point naturally.

### 8.1 Assessment of the probability density estimates

If the true probability density *f*_*X*_ is known a commonly used measure of error of a density estimate ${\hat{f}}_{X}$ is the mean integrated squared error (MISE):

$$\begin{array}{c} \text{MISE}=E_{f_{X}}\int_{D_{X}}{\left[{\hat{f}}_{X}\left(x\right)-f_{X}\left(x\right)\right]}^{2}\;dx \end{array}$$ (63)

We also adopt this metric here. The expectation with respect to *f*_*X*_ is evaluated as the mean over 100 simulations.

Often there is particular interest in detecting the large-scale structure of a probability density function as characterised by the number of modes or bumps [1] as these may be associated with distinct dynamical states or regimes of the underlying system (for example, [55, 56]). A mode is defined as a local maximum of the density; a bump is characterised by an interval on which the density has negative curvature but not necessarily a local maximum. As opposed to [56] we here refer to the curvature of the density itself and not the curvature of the potential function. The number of bumps is always larger or equal to the number of modes.

### 8.2 Simulated data

We start the investigation with simulated data sets where the underlying probability density is known. The test cases are listed in Table 3. Many of them are taken from [59]; some are slightly modified. Table 4 gives the MISE for the various density estimators and different sample sizes *N*. Table 5 lists for selected test cases how often the number of modes and bumps of the density is correctly identified.

**Table 3:** Test cases of probability densities: Densities 1–12 are supported on (−∞, ∞), density 13 on [−2, 2], density 14 on [0, 1] and density 15 on [0, ∞). *N*(*μ*, *σ*^2^) denotes the normal density with mean *μ* and standard deviation *σ*, Beta(*α*, *β*) denotes the beta density with shape parameters *α* and *β*, and Gam(*α*, *β*) denotes the gamma density with shape parameter *α* and rate parameter *β*.

Test case	Description	Probability density function *f*_*X*_(*x*)
1	normal	*N*(0, 1)
2	strongly skewed unimodal	$\frac{1}{8}\sum_{k=0}^{7}N(3[{\left(\frac{2}{3}\right)}^{k}-1],{\left(\frac{2}{3}\right)}^{2k})$
3	kurtotic unimodal	$\frac{2}{3}N\left(0,1\right)+\frac{1}{3}N(0,{\left(\frac{1}{10}\right)}^{2})$
4	outlier unimodal	$\frac{1}{10}N\left(0,1\right)+\frac{9}{10}N(0,{\left(\frac{1}{8}\right)}^{2})$
5	unimodal, 2 bumps	$\frac{13}{20}N\left(0,1\right)+\frac{7}{20}N\left(\frac{5}{2},1\right)$
6	skewed bimodal	$\frac{3}{4}N\left(0,1\right)+\frac{1}{4}N(\frac{3}{2},{\left(\frac{1}{3}\right)}^{2})$
7	separated bimodal	$\frac{1}{2}N(-2,{\left(\frac{1}{2}\right)}^{2})+\frac{1}{2}N(2,{\left(\frac{1}{2}\right)}^{2})$
8	asymmetric bimodal	$\frac{1}{2}N(0,{\left(\frac{1}{5}\right)}^{2})+\frac{1}{2}N\left(5,1\right)$
9	trimodal	$\frac{1}{3}\sum_{k=0}^{2}N(80k,{\left(k+1\right)}^{4})$
10	5 modes	$\frac{1}{5}\sum_{k=0}^{4}N(80k,{\left(k+1\right)}^{2})$
11	claw	$\frac{1}{2}N\left(0,1\right)+\frac{1}{10}\sum_{k=0}^{4}N(\frac{k}{2}-1,{\left(\frac{1}{10}\right)}^{2})$
12	asymmetric claw	$\frac{1}{2}N\left(0,1\right)+\sum_{k=-2}^{2}\frac{2^{1-k}}{31}N(k+\frac{1}{2},{\left(\frac{2^{-k}}{10}\right)}^{2})$
13	skewed bimodal	Test case 6 truncated to [−2, 2]
14	asymmetric bimodal	$\frac{1}{2}\text{Beta}\left(2,7\right)+\frac{1}{2}\text{Beta}\left(6,4\right)$
15	asymmetric bimodal	$\frac{4}{5}\text{Gam}\left(2,2\right)+\frac{1}{5}N\left(2,0.15^{2}\right)$ truncated to [0, ∞)

**Table 4:** Mean integrated squared error (scaled by 10^4^) estimated as the average over 100 realisations for the test cases in Table 3. For test cases 14 and 15, the semiparametric estimators allow for logarithmic boundary terms at any boundary point. The best method is indicated in bold. For test cases 9 and 10, the diffusion estimators are unstable; for test cases 4, 8 and 11, they give reasonable density estimates in most realisations but are unstable in some realisations. See S3 Appendix for further explanation.

Test case	*N*	KDE1/2/3	DE0/1	POL	SPL1/2	TRIG	SPDE	LLDE0/1/2
1	500	17/16/17	16/11	**5.0**	8.3/8.4		**5.0**	18/10/8.1
2	1000	750/632/104	247/203	131	158/82		**79**	218/249/152
2	5000	475/372/36	54/41	77	71/**22**		**22**	81/75/58
3	1000	304/417/80	160/109	431	142/**56**		**56**	304/289/159
3	5000	123/187/33	60/37	246	32/25		**23**	109/94/45
4	1000	82/79/108	(400)/(236)	82	**69**/282		81	1028/503/108
4	5000	**26**/**26**/62	(112)/(77)	51	31/93		31	226/97/32
5	1000	8.3/9.7/8.9	8.8/**7.5**	13	10/10		8.9	10/11/10
5	10000	1.5/1.9/1.6	2.3/**1.2**	2.1	2.8/1.8		1.8	2.2/2.9/2.2
6	1000	24/45/20	20/**18**	33	33/22		20	26/31/24
6	10000	5.0/12/3.2	4.5/**2.3**	5.1	4.0/4.9		3.6	5.1/6.4/5.5
7	500	256/592/35	32/**26**	38	36/89		37	39/**26**/27
7	5000	68/280/6.1	8.1/4.4	6.0	**3.6**/17		**3.6**	7.7/4.9/4.5
8	1000	1623/1735/52	(513)/(458)	38	35/71		35	93/86/**17**
8	5000	1134/1322/14	(93)/(51)	7.4	4.5/25		4.5	28/27/**3.5**
9	1000	322/373/8.9		**3.2**	7.5/11		3.8	36/24/4.0
9	10000	282/343/1.8		**0.3**	0.9/3.0		0.5	3.7/4.0/0.4
10	1000	219/227/6.8		**2.9**	14/10		**2.9**	8.9/9.4/3.1
10	10000	196/208/1.2		**0.4**	1.7/2.6		**0.4**	2.0/2.2/**0.4**
11	1000	393/436/**69**	(258)/(183)	511	118/89		86	147/170/104
11	10000	206/293/**12**	(79)/(53)	396	154/20		20	24/23/14
12	1000	159/202/76	118/80	129	104/170		104	84/80/**72**
12	10000	86/122/15	53/39	61	51/57		51	17/16/**12**
13	1000	29/50/25	25/27	38	31/**23**	29	26	130/77/103
13	10000	6.8/16/4.1	3.7/3.6	5.5	**2.7**/3.1	4.3	**2.7**	28/18/28
14	1000	155/271/128	138/138	80	74/78	**63**	70	218/161/280
14	5000	63/138/37	43/39	22	21/**17**	27	26	96/46/73
15	1000	126/238/**47**	54/67	95	61/62		58	80/68/80
15	5000	56/125/**14**	19/19	69	25/17		16	31/28/22

**Table 5:** Detection of modality: Number of realisations out of 100 in which the modality of the density is correctly identified. The upper row refers to modes, the lower row to bumps. Modes and bumps are counted on the indicated interval. For test cases 14 and 15, the semiparametric estimators allow for logarithmic boundary terms at any boundary point. The best method is indicated in bold. For test case 8, the diffusion estimators give reasonable density estimates in most realisations but are unstable in some realisations. See S3 Appendix for further explanation.

Test case	*N*	Interval	KDE1/2/3	DE0/1	POL	SPL1/2	TRIG	SPDE	LLDE0/1/2
1	500	[−5.5, 5.5]	56/75/34	85/95	**100**	**100**/**100**		**100**	82/18/65
			0/0/4	0/3	**100**	**100**/**100**		**100**	0/0/65
1	500	[−3.5, 3.5]	72/89/94	86/99	**100**	**100**/**100**		**100**	89/98/98
			0/0/20	8/75	**100**	**100**/**100**		**100**	30/84/94
2	1000	[−4, 4]	16/4/0	0/0	3	89/**100**		**100**	0/0/0
			0/0/0	0/0	0	59/**78**		76	0/0/0
2	5000	[−4, 4]	26/8/0	0/0	16	96/**100**		**100**	0/0/0
			0/0/0	0/0	0	71/**92**		**92**	0/0/0
5	1000	[−4, 6]	59/90/68	44/84	99	**100**/**100**		**100**	42/71/78
			1/18/20	16/84	55	**99**/95		96	21/53/65
5	10000	[−4, 6]	56/86/51	4/69	**100**	99/99		99	44/65/68
			0/23/0	0/38	**100**	**100**/**100**		**100**	23/40/61
6	1000	[−5.5, 5.5]	67/79/0	35/61	57	51/**81**		**81**	39/1/0
			0/0/0	0/1	32	92/**98**		97	0/0/0
6	1000	[−3.5, 3.5]	84/**87**/39	36/78	66	51/81		81	46/49/54
			0/13/0	0/4	37	92/**98**		97	5/11/29
6	10000	[−3.5, 3.5]	86/**100**/16	1/59	71	98/**100**		98	45/51/73
			0/28/0	0/0	4	64/**100**		79	3/6/30
7	500	[−4, 4]	**100**/**100**/83	86/99	**100**	**100**/92		**100**	62/1/39
			**100**/38/8	18/75	73	96/42		95	17/1/1
8	1000	[−0.5, 8]	**100**/95/0	(0)/(0)	94	98/95		98	1/0/0
			33/37/0	(0)/(0)	30	**83**/48		82	0/0/0
13	1000	[−2, 2]	80/**93**/48	71/77	75	76/76	84	80	0/27/13
			0/0/0	0/2	64	98/**100**	32	87	0/13/2
13	10000	[−2, 2]	83/**100**/14	44/57	**100**	**100**/**100**	**100**	**100**	0/15/2
			0/0/0	0/0	50	**100**/**100**	13	**100**	0/0/0
14	1000	[0, 1]	83/99/49	60/76	92	**100**/**100**	97	98	0/12/0
			0/0/0	0/1	**100**	99/99	**100**	**100**	0/4/0
14	5000	[0, 1]	79/99/8	4/33	**100**	**100**/**100**	**100**	**100**	0/0/0
			0/0/0	0/0	99	98/**100**	**100**	**100**	0/0/0
15	1000	[0, 4]	6/32/0	0/0	3	43/**77**		70	0/0/0
			0/0/0	0/0	2	8/**41**		32	0/0/0
15	5000	[0, 4]	0/19/0	0/0	2	51/**99**		93	2/0/0
			0/0/0	0/0	1	10/**80**		65	0/0/0

The density estimator SPDE has an excellent overall performance in the MISE. It is sometimes the best model and is always close to the best model, except for test case 12. At the same time, the SPDE model exhibits a high degree of global smoothness which enables a top performance in modality detection. It often has the highest detection rate and is always close to the top model. The number of modes and bumps depends on the interval over which they are counted. The interval is chosen by eye around the main body of the distribution but also an extension further out into the tails was investigated. The estimators SPL1, SPL2, TRIG, SPDE and also POL in those cases where it is an appropriate model are very robust with respect to the choice of the interval and are thus adequate for fully automatic detection of modes and bumps. All of the other estimators except finite mixture models often have very small spurious modes and bumps far out in the tails.

In some cases the MISE of SPDE is smaller than the smallest among the four components POL, SPL1, SPL2 and TRIG as it picks the best for each of the 100 realisations. Often it is equal to or slightly larger than the smallest among the components. This is probably due to the different metrics used here. The density models are fitted with maximum likelihood but validated with the MISE. When evaluated in terms of Kullback–Leibler divergence the error of SPDE would probably be at least as small as the smallest among the components.

Polynomials and splines work complementarily in the semiparametric method. Polynomials perform well for well separated and not very sharp structures (test cases 1, 9 and 10); on the other hand splines are much better for overlapping and/or sharp structures (test cases 2, 3, 11 and 15) as well as long tails with little structure (test case 2). This is in line with approximation theory; put in statistical terms the density is in the latter case not well chracterised by (global) polynomial moments but local moments are much more informative. In many test cases polynomials and splines perform about equally well. Both spline knot placement schemes considerably contribute to the excellent overall performance of SPDE. We generally see that asymptotic convergence rates are not necessarily relevant at finite sample sizes. Splines have a slower convergence rate than polynomials and trigonometric functions (see Subsection 2.4); yet, in most test cases they show a smaller MISE.

Among the kernel density estimators KDE3 vastly outperforms KDE1 and KDE2 in terms of the MISE as the latter two estimators almost always oversmooth the data. They are only competitive for densities relatively close to a Gaussian (test cases 1, 4, 5 and 6). The reason is that the bandwidth selection of KDE1 and KDE2 is based on the normal reference rule whereas that of KDE3 is not [7]. On the other hand, KDE1 and KDE2 are often able to robustly detect the modality of the density whereas KDE3 is very bad at it as it easily develops spurious modes and bumps, particularly for long tails (test case 2) and densities with features of different scales (test case 8). There seems to be no way out of this dilemma as long as a non-adaptive bandwidth is used. The ability of KDE3 to detect the modality sometimes even becomes worse when increasing the sample size, in contrast to KDE1 and KDE2. We remark that the behaviour of the kernel density estimator with bandwidth given by the classical Sheather–Jones solve-the-equation rule [5] (not shown) is somewhat in between KDE1/2 and KDE3. In line with the findings in [7], it has a larger MISE than KDE3, particularly for bi- and multimodal densities, but the modality detection is better and more robust.

Among the diffusion estimators DE1 is here found to consistently outperform DE0, both in terms of MISE and detection of the modality of the density. The estimator DE1 improves on KDE3 regarding modality detection by adding some smoothness. Remarkably, at the same time it often also improves the MISE and is never much worse. However, DE1 does not reach the smoothness of the semiparametric estimators.

The model LLDE2 is usually the best among the local likelihood density estimators with some exceptions. It shows some very good and even top performances with localised structures, though there may be some bias here as the structures are Gaussian which exactly fits the locally quadratic model. But LLDE2 does not possess the broad strength of SPDE; in particular it is lacking global smoothness which shows up in some very poor modality detection performances.

Expectedly, a Gaussian mixture model (GMM) outperforms all of the other methods in terms of both the MISE and modality detection for almost all test cases (not shown) as these are given as Gaussian mixtures. It is worth noting that this is not the case if the true number of components is too high to be identified given the sample size (test case 2). Moreover, the likelihood of a GMM with many components is prone to have plenty of secondary maxima and therefore it sometimes takes very many random initialisations of the EM algorithm to actually find the correct model. It is also interesting that for test case 5, which is a mixture of two Gaussians, the estimator SPDE outperforms a GMM with two components in terms of bump detection.

A (rescaled) beta mixture model performs worse than the SPDE for test case 13 (not shown) and it is obviously biased for test case 14. For test case 15, a gamma and a Weibull mixture model perform slightly worse than the SPDE (not shown).

**Fig 1:**
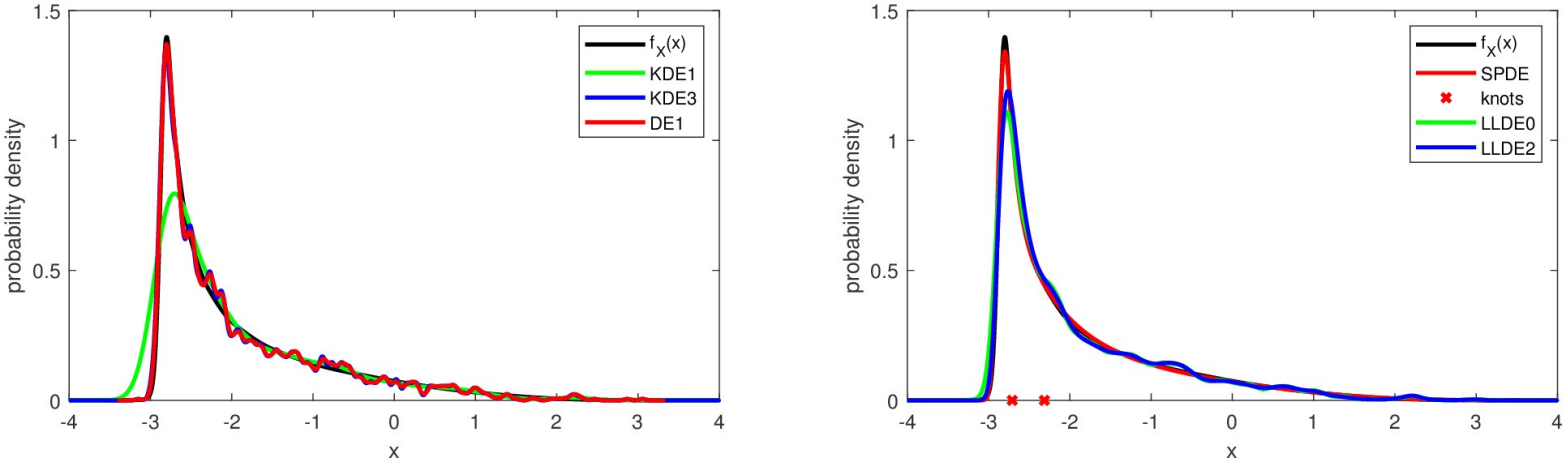
Test case 2: Example of density estimates obtained from the kernel density and diffusion estimators (left) as well as from the semiparametric and local likelihood estimators (right). The SPDE is the model SPL2 with 2 knots after knot deletion. The sample size is *N* = 2000.

**Fig 2:**
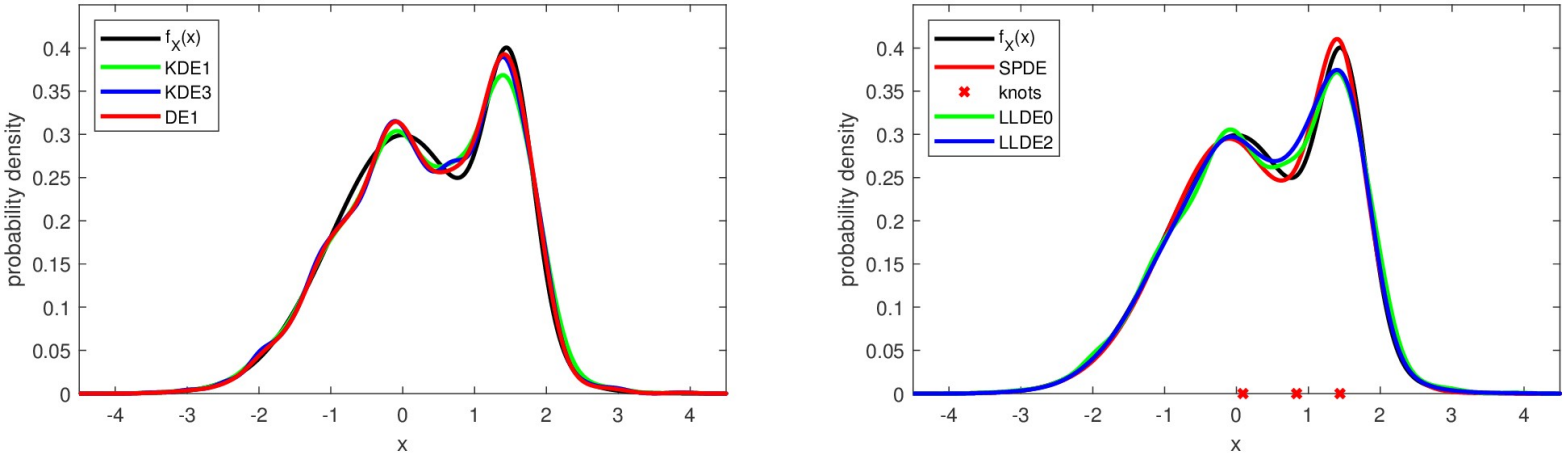
Test case 6: Example of density estimates obtained from the kernel density and diffusion estimators (left) as well as from the semiparametric and local likelihood estimators (right). The SPDE is the model SPL2 with 3 knots after knot deletion. The sample size is *N* = 2000.

The points made above are illustrated in Figs 1 and 2 with typical density estimates from the various methods for the test cases 2 and 6. The SPDE in both cases is the model SPL2 with 2 and 3 knots, respectively, after knot deletion.

**Fig 3:**
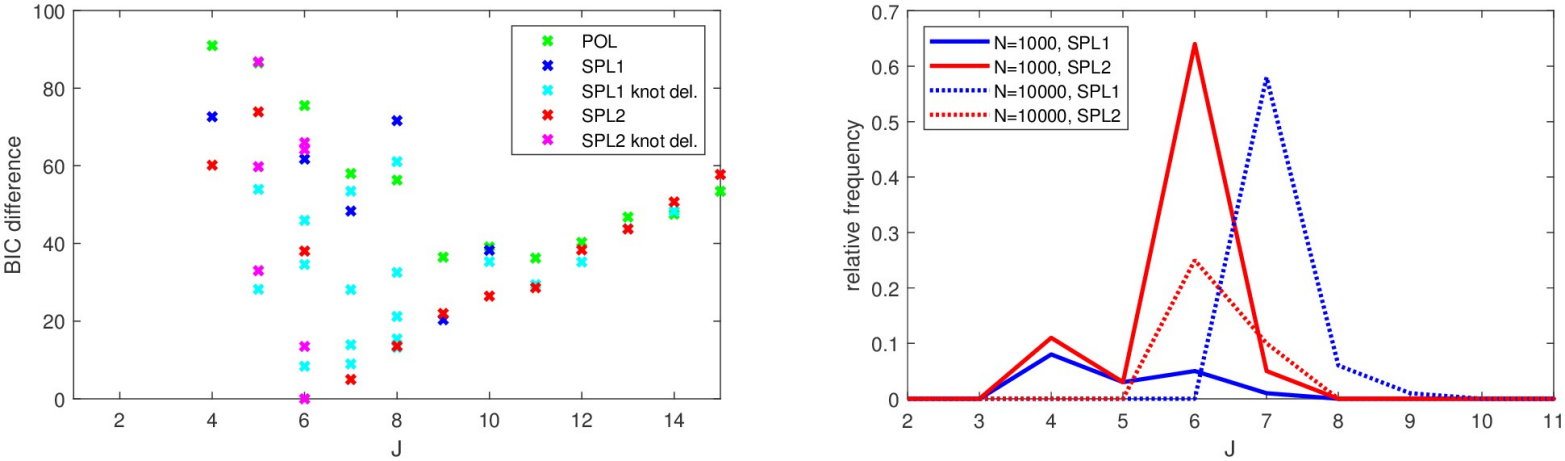
Test case 6: Left: Example of model selection, corresponding to the example shown in Fig 2. Sample size is *N* = 2000. Right: Relative frequency out of 250 realisations of picking a particular model as the SPDE. The spline models encompass models without and with knot deletion.

Fig 3 illustrates an example (corresponding to Fig 2) and the summary statistics of model selection for test case 6. The plot indicates all of the models considered, including every single candidate spline model encountered during the knot deletion procedure. Only BIC differences are given with the best model put at zero. Only spline models are used, both SPL1 and SPL2. The model complexity increases rather slowly with the sample size.

Some more examples of density estimates and model selections are displayed in S4 Appendix, including cases involving rather high-dimensional models. Some test cases make use of all of the estimators POL, SPL1 and SPL2, dependent on the sample size. Substantial gains from knot deletion are seen for the spline models. The order of polynomial models tends to increase faster with sample size than the order of spline models. For test case 4, there is a slight indication that the polynomial model is picked increasingly often at very large sample sizes, maybe due to its superior asymptotic convergence rate.

The results discussed above equally hold for bounded, infinite and semi-infinite domains. We now look at the boundary effects in test cases 13, 14 and 15. The density of test case 13 is bounded away from zero over the whole domain and also at both boundary points *x* = −2 and *x* = 2. Therefore boundary terms are not useful here. Test case 14 has zero density with finite slope at *x* = 0 and zero density with zero slope at *x* = 1; test case 15 has zero density with finite slope at *x* = 0. For these two test cases we consider logarithmic boundary terms at all of the boundary points. In order to assess the effects of the boundary terms we also model these two cases with all boundary terms switched off.

Tables 6 and 7 display how often which boundary terms are included in the model for test cases 14 and 15, respectively. Boundary terms are chosen at all of the boundary points in a substantial fraction of realisations; the frequency increases with the sample size. For test case 14, there is slightly more need for a boundary term at *x* = 0 than at *x* = 1 as due to the finite slope the boundary bias is larger there. We observe some differences among the various semiparametric density estimators regarding the frequency of including certain boundary terms.

**Table 6:** Test case 14: Number of realisations out of 100 in which certain combinations of logarithmic boundary terms are chosen for the various semiparametric estimators and different sample sizes.

Model	POL	SPL1	SPL2	TRIG	SPDE
Sample size *N*	1000/5000	1000/5000	1000/5000	1000/5000	1000/5000
No boundary terms	14/1	55/3	51/1	13/4	23/1
Only left boundary term	15/75	41/63	36/54	2/5	5/28
Only right boundary term	0/2	2/2	9/7	0/0	0/3
Left+right boundary terms	71/22	2/32	4/38	85/91	72/68

**Table 7:** Test case 15: Number of realisations out of 100 in which no boundary term or a logarithmic boundary term is chosen for the various semiparametric estimators and different sample sizes.

Model	POL	SPL1	SPL2	SPDE
Sample size *N*	1000/5000	1000/5000	1000/5000	1000/5000
No boundary term	88/49	53/13	55/14	52/11
Left boundary term	12/51	47/87	45/86	48/89

Table 8 contrasts the MISE of the different semiparametric methods without and with boundary terms for test cases 14 and 15. There is a substantial improvement for test case 14 and a small but systematic improvement for test case 15 due to the boundary terms. Also a moderate systematic increase of the probability of correct detection of the number of modes and bumps in the densities can be observed when allowing for boundary terms (not shown).

**Table 8:** Effect of boundary terms on model accuracy: Mean integrated squared error (scaled by 10^4^) estimated as an average over 100 realisations. The upper row refers to models without boundary terms, the lower row to models allowing for logarithmic boundary terms at any boundary point.

Test case	*N*	POL	SPL1/2	TRIG	SPDE
14	1000	105	101/104	96	93
		80	74/78	63	70
14	5000	27	37/38	34	36
		22	21/17	27	26
15	1000	92	65/63		62
		95	61/62		58
15	5000	68	25/19		18
		69	25/17		16

Table 9 lists the mean boundary biases of the various estimators. The semiparametric techniques, already without boundary terms, exhibit significantly reduced boundary biases compared to the kernel density and diffusion estimators. The diffusion estimators are actually consistent at any boundary point as *N* → ∞ [7] but convergence is very slow. The inclusion of boundary terms greatly reduces the boundary biases of the semiparametric estimators. This is particularly pertinent at large sample size. The local likelihood density estimators in some cases also have very low boundary biases, though this is achieved by the choice of a rather small bandwidth which leads to very wiggly density estimates and the inability to detect the modality of the densities. For the cases with large boundary biases a pronounced improvement is seen when increasing the order of the local model. This is in accordance with the theory of local likelihood density estimation [16, 17] which expands the boundary bias into powers of the bandwidth and predicts an improvement of the order of accuracy by one when increasing the order of the local model by one. For small boundary biases this effect is overlaid with the sampling uncertainty in only 100 realisations. Overall, the semiparametric estimators in all cases of Table 9 either perform best or are close to the best model within the sampling uncertainty.

**Table 9:** Boundary bias (scaled by 10^4^) estimated as the mean over 100 realisations. The true density at the boundary is *f*_*X*_(−2) = 0.0427 and *f*_*X*_(2) = 0.1450 for test case 13, *f*_*X*_(0) = *f*_*X*_(1) = 0 for test case 14, and *f*_*X*_(0) = 0 for test case 15. For the semiparametric estimators and test cases 14 and 15, the upper row refers to models without boundary terms and the lower row to models allowing for logarithmic boundary terms at any boundary point. The best method is indicated in bold.

Test case	*N*	*x*	KDE1/2/3	DE0/1	POL	SPL1/2	TRIG	SPDE	LLDE0/1/2
13	1000	-2	171/289/124	145/103	144	**1**/-35	-122	-15	6/75/-51
13	1000	2	1081/1438/837	850/898	221	225/51	203	60	**31**/263/-235
14	1000	0	7990/9958/6621	6508/6909	4115	4085/3969	3734	3652	2784/2195/917
					541	2110/2182	**475**	772	
14	1000	1	357/957/186	365/185	55	106/109	70	107	11/21/**10**
					30	96/100	13	34	
14	5000	0	6410/8444/4380	4293/4487	2468	2989/2849	2803	2765	2006/1153/594
					**69**	139/228	122	95	
14	5000	1	162/462/44	105/43	22	36/80	46	40	5/**2**/4
					40	49/55	10	23	
15	1000	0	2879/3496/1740	1407/1826	483	981/806		897	1306/905/422
					**371**	517/496		491	
15	5000	0	2308/2919/1206	926/1257	533	741/549		576	1112/752/347
					177	99/83		**67**	

**Fig 4:**
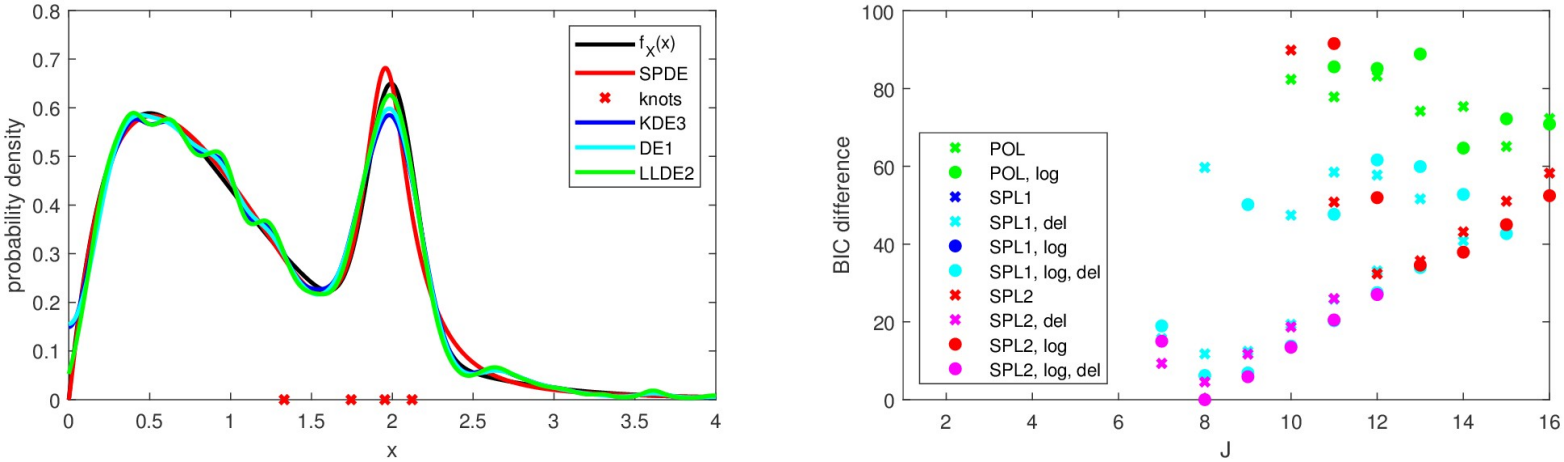
Test case 15: Example of density estimates from selected methods (left) and corresponding model selection for the SPDE (right). Only the best spline model after each knot deletion step is indicated to increase the readability of the plot. The SPDE is the model SPL2 with 4 knots after knot deletion augmented with a logarithmic boundary term. The sample size is *N* = 2000.

Fig 4 displays for test case 15 a couple of typical density estimates from selected methods and the corresponding model selection for the SPDE. The SPDE here is the model SPL2 with 4 knots after extensive knot deletion and a logarithmic boundary term. For this test case, knot deletion delivers a particularly huge gain in BIC over spline models without knot deletion. The lack of boundary bias and again the overall smoothness of the SPDE are clearly visible.

The computation time of the various techniques depends in a complicated manner on the sample size, the model complexity and the implementation details. A couple of very general comments are in order. The kernel density estimators are more than an order of magnitude faster than all of the other methods with KDE3 being particularly fast. The semiparametric techniques and the diffusion estimators are about equally costly. The full model selection of the semiparametric estimator for a data sample of test case 15 as illustrated in Fig 4 involving quite high-dimensional models, a boundary term and extensive knot deletion requires still just about a second on a PC. The finite mixture models and the local likelihood estimators are significantly more expensive than the semiparametric methods (by a factor of 3–20, depending on the sample size and the model complexity).

### 8.3 Old Faithful geyser data

**Fig 5:**
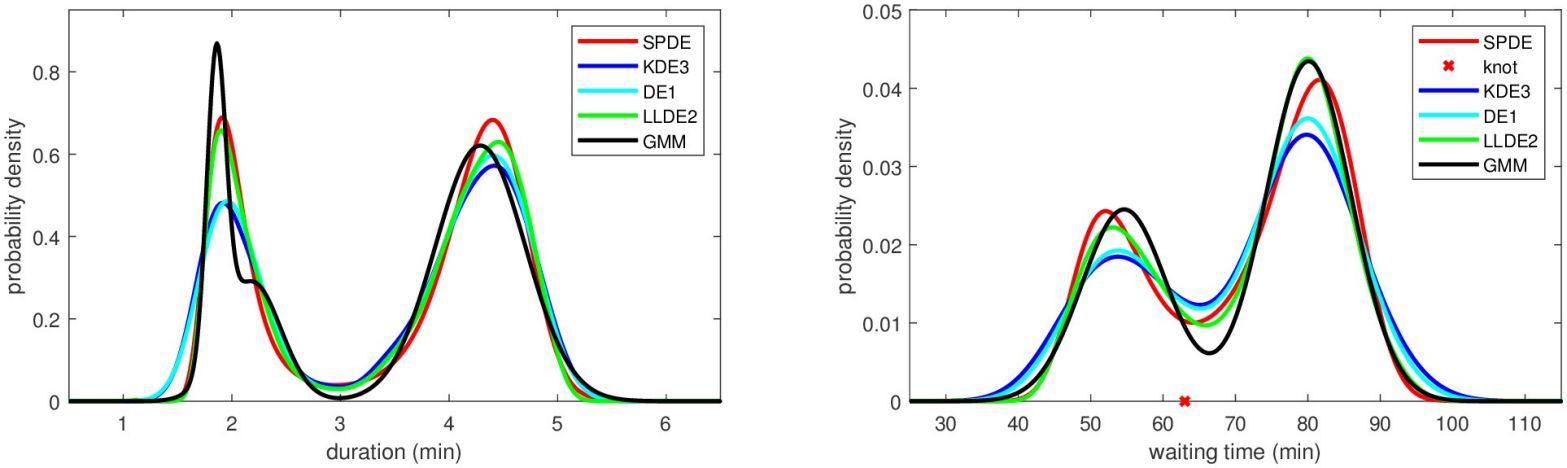
Old Faithful Geyser eruption data: Density estimates of the eruption duration (left) and waiting time (right) from various methods. For the duration data the SPDE is the model with potential function *U*(*y*) = *α*_1_log *y* + *α*_2_(1/*y*) + *α*_3_ *y* + *α*_4_ *y*^2^; for the waiting time data it is the model SPL2 with 1 knot after knot deletion.

As an example of observational data we investigate the Old Faithful geyser data. There are data sets of the eruption duration and of the waiting time between successive eruptions of the Old Faithful geyser in Yellowstone National Park, Wyoming, USA, both consisting of *N* = 272 observations. Both data sets are supported on the semi-infinite interval [0, ∞) and the option of all subsets of the boundary terms {log *y*, 1/*y*} is included. As the true underlying densities are unknown here the leave-one-out cross-validation likelihood (cvlogl) is used for model validation. Table 10 lists an overview of the results for all of the density estimation techniques and Fig 5 displays the density estimates for a couple of selected methods.

**Table 10:** Old Faithful geyser data: Bayesian information criterion, cross-validated log-likelihood, number of modes and number of bumps for various density estimators. The number of modes and bumps is counted on the interval [1.25, 5.5] for the eruption duration data and on the interval [35, 100] for the waiting time data. The best method is indicated in bold.

	Duration				Waiting time			
	BIC	cvlogl	Modes	Bumps	BIC	cvlogl	Modes	Bumps
KDE1		-302.0	2	2		-1044.4	2	2
KDE2		-300.8	2	2		-1045.0	2	3
KDE3		-276.9	2	2		-1049.3	2	2
DE0		-275.8	2	2		-1047.2	2	2
DE1		-275.1	2	2		-1044.3	2	2
GMM	572.7	-274.2	3	3	2096.0	-1038.9	2	2
POL	**544.0**	**-264.5**	2	2	2097.9	-1039.9	2	2
SPL1	548.2	-266.4	2	2	2093.5	-1039.6	2	2
SPL2	546.9	-265.8	2	2	**2093.3**	-1039.5	2	2
LLDE0		-270.9	3	9		-1040.1	2	5
LLDE1		-268.5	3	7		-1039.3	2	2
LLDE2		-267.2	2	2		**-1038.6**	2	2

For the eruption duration data the estimators POL, SPL1 and SPL2 perform best and robustly detect 2 modes and bumps; LLDE2 is almost as good. The SPDE is the model POL with boundary terms; it has potential function *U*(*y*) = *α*_1_ log *y* + *α*_2_(1/*y*) + *α*_3_ *y* + *α*_4_ *y*^2^ which is a generalised inverse Gaussian distribution augmented with the quadratic term to generate the bimodality. Working without boundary terms a sixth-order polynomial model ($U\left(y\right)=\sum_{j=1}^{6}\alpha_{j}y^{j}$) is only slightly worse in terms of cross-validated likelihood and still better than all of the other density estimators. The spline estimators SPL1 and SPL2 have no boundary terms and a single knot, SPL2 after knot deletion. The estimators KDE1, KDE2 and KDE3 as well as DE0 and DE1 perform significantly worse as they oversmooth the data; this is particularly true for KDE1 and KDE2. Also a Gaussian mixture model (GMM) (truncated to [0, ∞) after the inference) does not perform well as both modes of the density are strongly non-Gaussian. The BIC picks a model with 3 components which detects a spurious third mode in the density. Also a gamma mixture model (GaMM) and a Weibull mixture model (WMM) are not appropriate here. The estimators LLDE0 and LLDE1 are quite wiggly and display small spurious modes and bumps.

For the waiting time data all of the semiparametric models have no boundary terms. The estimator POL is a fifth-order polynomial model ($U\left(y\right)=\sum_{j=1}^{5}\alpha_{j}y^{j}$); the estimators SPL1 and SPL2 both have 1 knot after knot deletion. The model selected by the BIC is SPL2. The estimators POL, SPL1/2, LLDE0/1/2 and GMM with 2 components (truncated to [0, ∞) after the inference) perform well in terms of cross-validated likelihood with LLDE2 being best; KDE1/2/3 and DE0/1 are worse. The mixture models GaMM and WMM are worse than GMM. All of the estimators detect two modes which are both quite close to Gaussian. The models KDE2 and LLDE0 have spurious bumps, all of the others detect two bumps.

## 9 Conclusions

A methodology for semiparametric maximum likelihood probability density estimation was discussed. The density is represented by sequences of exponential families generated by flexible sets of basis functions including polynomials, trigonometric functions and splines with two different knot placement schemes and a knot deletion scheme, optionally augmented with logarithmic and rational boundary terms. A BIC model selection over the number as well as the type of basis functions is implemented. The technique is explored on a host of simulation and observation data sets and compared to a couple of common density estimators. The test cases include uni-, bi- and multi-modal densities, Gaussian and non-Gaussian modes, features of different scales in a density and long tails without structure on bounded, infinite and semi-infinite intervals. Unlike all of the other methods, the SPDE consistently shows an excellent overall performance taking into account the MISE/cvlogl, detection of modality and computation time. It combines a very small MISE or large cvlogl with a high degree of global smoothness which enables the correct detection of the large-scale structure of the density in terms of the number of modes and bumps. All of the types of bulk basis functions as well as the boundary terms significantly contribute to the practical performance of the SPDE.

An extension of the method to the multivariate case is straightforward. One would use multinomials [37, 38], tensor-product splines [60] or multiple Fourier series as basis functions which account for cross-variable dependencies in a natural way. Boundary terms could be added where appropriate and model selection performed as in the univariate case.

A comparison of the ranking of the different density models in terms of the BIC and the cross-validated likelihood for the Old Faithful geyser data sets reveals some limitations of the use of the BIC for model selection. The BIC is reliable in comparing models with the same type of basis functions and it is still about adequate to compare polynomial models with spline or trigonometric models. However, when including models with boundary terms and finite mixture models in the comparison there are cases where two models differ by about 6 points in the BIC but have about the same cross-validated likelihood. Ideally, the model selection should be based on some cross-validated score or even an out-of-sample score in data-rich settings. This is often computationally infeasible; if the order of the appropriate model is relatively low 10-fold cross-validation, for example, may still be practical.

## Supporting information

S1 Appendix Existence of the maximum likelihood estimator. (PDF)

S2 Appendix The truncated moment problem and the moment-constrained maximum entropy principle. (PDF)

S3 Appendix Alternative methods for density estimation. (PDF)

S4 Appendix Further results. (PDF)
