# Supplementary material for: Semiparametric maximum likelihood probability density estimation
Source: PLoS One. 2021 Nov 9;16(11):e0259111. doi: 10.1371/journal.pone.0259111 (PMC8577774; doi:10.1371/journal.pone.0259111)
Supplement: S1 Appendix — (PDF) [file pone.0259111.s001.pdf]

Supporting Information – S1 Appendix

Semiparametric maximum likelihood probability density estimation

Frank Kwasniok

Existence of the maximum likelihood estimator

The standard results on the existence of the maximum likelihood estimator for exponential families [1, 2, 3] are briefly summarised. They are then applied to all of the domains and sets of basis functions considered here which include non-standard settings not considered in the literature so far.

Definition: A minimal and full exponential family of order J is regular if the parameter space

$$\mathcal{P} = \{\boldsymbol{\alpha} \in \mathbb{R}^J \,|\, Z(\boldsymbol{\alpha}) < \infty\}$$

is an open set, that is, if $\mathcal{P} = \operatorname{int}\mathcal{P}$.

Definition: A minimal and full exponential family is steep if for any $\boldsymbol{\alpha}_\mathrm{b} \in \mathcal{P}\backslash\operatorname{int}\mathcal{P}$ and any $\boldsymbol{\alpha}_\mathrm{int} \in \operatorname{int}\mathcal{P}$

$$\lim_{\tau\uparrow 1} \frac{\partial}{\partial\tau} A(\boldsymbol{\alpha}_\mathrm{int} + \tau(\boldsymbol{\alpha}_\mathrm{b} - \boldsymbol{\alpha}_\mathrm{int})) = \lim_{\tau\uparrow 1} (\boldsymbol{\alpha}_\mathrm{b} - \boldsymbol{\alpha}_\mathrm{int})^\mathrm{T} \nabla_{\boldsymbol{\alpha}} A(\boldsymbol{\alpha}_\mathrm{int} + \tau(\boldsymbol{\alpha}_\mathrm{b} - \boldsymbol{\alpha}_\mathrm{int})) = \infty$$

with $0 \leq \tau < 1$. Any regular exponential family is steep as there are no boundary points in $\mathcal{P}$.

The support of the sufficient statistics is a subset of $\mathbb{R}^J$ given by

$$\Gamma = \left\{\boldsymbol{\phi}(y) = (\phi_1(y), \ldots, \phi_J(y))^\mathrm{T} \,|\, y \in D\right\}, \tag{1}$$

and the closed convex hull of Γ is $\mathcal{X} = \overline{\operatorname{conv}\Gamma}$. The range of the gradient of the log-partition function is

$$\mathcal{R} = \{\mathbf{m}(\boldsymbol{\alpha}) \,|\, \boldsymbol{\alpha} \in \operatorname{int}\mathcal{P}\}. \tag{2}$$

Maximum likelihood inference in exponential families is characterised by the following theorem [1]:

Theorem 1 *For a minimal, full and steep exponential family the following statements hold: The function $\mathbf{m}(\boldsymbol{\alpha})$ is real analytic and bijective between $\operatorname{int}\mathcal{P}$ and $\mathcal{R} = \operatorname{int}\mathcal{X}$. The likelihood function attains its maximum if and only if $\langle\boldsymbol{\phi}\rangle \in \operatorname{int}\mathcal{X}$ and the maximum likelihood estimator is then given by the unique solution of the likelihood equations, $\hat{\boldsymbol{\alpha}} = \mathbf{m}^{-1}(\langle\boldsymbol{\phi}\rangle) \in \operatorname{int}\mathcal{P}$.*

For a minimal and full exponential family which is not steep the following statements hold: The function $\mathbf{m}(\boldsymbol{\alpha})$ is real analytic and bijective between $\operatorname{int}\mathcal{P}$ and $\mathcal{R} \subset \operatorname{int}\mathcal{X}$ with $\operatorname{int}\mathcal{X}\backslash\mathcal{R}$ being non-empty. The likelihood function attains its maximum if and only if $\langle\boldsymbol{\phi}\rangle \in \operatorname{int}\mathcal{X}$. If $\langle\boldsymbol{\phi}\rangle \in \mathcal{R}$ the maximum likelihood estimator is given by the unique solution of the likelihood equations, $\hat{\boldsymbol{\alpha}} = \mathbf{m}^{-1}(\langle\boldsymbol{\phi}\rangle) \in \operatorname{int}\mathcal{P}$; if $\langle\boldsymbol{\phi}\rangle \in \operatorname{int}\mathcal{X}\backslash\mathcal{R}$ the likelihood equations have no solution and the likelihood function attains its maximum on $\mathcal{P}\backslash\operatorname{int}\mathcal{P}$.

We obviously have $\langle \boldsymbol{\phi} \rangle \in \mathcal{X}$ for any data sample $\{y_1, \ldots, y_N\}$ with $N \geq 1$. A data sample is called *generic* if $\langle \boldsymbol{\phi} \rangle \in \operatorname{int} \mathcal{X}$. For a steep exponential family the maximum likelihood estimator uniquely exists for any generic data sample. The requirement of genericity never poses any relevant restrictions in practical applications for any of the domains of support and models considered here. If the set of functions $\{1, \phi_1, \ldots, \phi_J\}$ forms a *Chebyshev system*, that is, it satisfies the *Haar condition* then any data sample which contains at least $J+1$ distinct data points is generic [4]. This condition is sufficient but not necessary; other simple regularity conditions guarantee genericity of any data sample with very few distinct data points [4]. These criteria together with elementary theorems about Chebyshev systems cover all of the model settings discussed here, including logarithmic and rational boundary terms. Splines fall slightly outside of this framework as they may vanish on finite subintervals but, for a cubic spline, any data sample is generic as soon as it contains at least four distinct data points on any section of the spline [5, 6] which is still a weak requirement.

Proper maximum likelihood inference is unclear if the exponential family is not steep. The above theorem gives no information what the set $\operatorname{int} \mathcal{X} \backslash \mathcal{R}$ is. It may be large and pose a severe restriction; it may only concern extreme combinations of sample means of the basis functions which rarely occur in practice. Density estimates obtained as solutions of the likelihood equations are still useful; we will not consider densities on the boundary of the parameter space as these are somewhat indicative that the model is not adequate for the data under consideration.

On the bounded interval $D = [-1, 1]$ exponential families generated by polynomial, spline or trigonometric basis functions for any J are regular as we have $\mathcal{P} = \mathbb{R}^J$ for any set of bounded basis functions. The model is still regular when introducing one or both of the logarithmic boundary terms $\{\log(y+1), \log(1-y)\}$, either solely (beta distribution) or in conjunction with the other basis functions. The model is not regular but steep as soon as one or both of the rational boundary terms $\{1/(y+1), 1/(1-y)\}$ are introduced, either solely or in conjunction with the other basis functions, but any model with both the logarithmic and rational term at the same boundary is not steep.

On the infinite domain $D = (-\infty, \infty)$ models generated by polynomials require the largest power to be even to be normalisable. They are not steep as soon as they contain two different even powers; otherwise they are regular. Exponential families generated by linearly extrapolated polynomials (Section 4 of the main text) are regular and have no restriction on the powers except that the model with a single linear term is not allowed. Models based on linearly extrapolated cubic splines (see [6] and Section 4 of the main text) are regular.

On the semi-infinite interval $D = [0, \infty)$ models generated by polynomials are not steep as soon as they contain two or more terms; models with a single monomial term ($U(y) = \alpha_1 y^k, k \in \mathbb{N}$) are regular. This still holds when adding any subset of the basis functions $\{\log y, 1/y, \log^2 y\}$. The gamma distribution ($U(y) = \alpha_1 \log y + \alpha_2 y$), the Rayleigh distribution ($U(y) = \log y + \alpha_1 y^2$), the Nakagami distribution ($U(y) = \alpha_1 \log y + \alpha_2 y^2$), the Weibull distribution with known shape parameter ($U(y) = (k-1) \log y + \alpha_1 y^k$), the inverse gamma distribution ($U(y) = \alpha_1 \log y + \alpha_2 (1/y)$) and the log-normal distribution ($U(y) = \alpha_1 \log y + \alpha_2 \log^2 y$) are regular; the inverse Gaussian distribution ($U(y) = -(3/2) \log y + \alpha_1 (1/y) + \alpha_2 y$) is not regular but steep; the generalised inverse Gaussian distribution ($U(y) = \alpha_1 \log y + \alpha_2 (1/y) + \alpha_3 y$) is not steep. Exponential families generated by linearly extrapolated polynomials or cubic splines (Section 5 of the main text) are regular. The extrapolated models are still regular when adding the boundary term $\{\log y\}$; they are not regular but steep with $\{1/y\}$; they are not steep with the subsets of basis functions $\{\log y, 1/y\}$, $\{\log y, \log^2 y\}$ or $\{\log y, 1/y, \log^2 y\}$. Here, the boundary terms are also linearly extrapolated.

References

[1] Barndorff-Nielsen OE. Information and Exponential Families in Statistical Theory. Wiley; 1978.

[2] Brown LD. Fundamentals of Statistical Exponential Families with Applications in Statistical Decision Theory. Lecture Notes. Volume 9. Institute of Mathematical Statistics; 1986.

[3] Sundberg R. Statistical Modelling by Exponential Families. Cambridge University Press; 2019.

[4] Crain BR. Exponential models, maximum likelihood estimation, and the Haar condition. Journal of the American Statistical Association. 1976; 71:737–740.

[5] Barron AR, Sheu C-H. Approximation of density functions by sequences of exponential families. The Annals of Statistics. 1991; 19:1347–1369.

[6] Kooperberg C, Stone CJ. A study of logspline density estimation. Computational Statistics & Data Analysis. 1991; 12:327–347.
