# Supplementary material for: Semiparametric maximum likelihood probability density estimation
Source: PLoS One. 2021 Nov 9;16(11):e0259111. doi: 10.1371/journal.pone.0259111 (PMC8577774; doi:10.1371/journal.pone.0259111)
Supplement: S2 Appendix — (PDF) [file pone.0259111.s002.pdf]

Supporting Information – S2 Appendix

Semiparametric maximum likelihood probability density estimation

Frank Kwasniok

The truncated moment problem and the moment-constrained maximum entropy principle

The truncated moment problem addresses the question whether a given sequence of real numbers $(\mu_0, \mu_1, \ldots, \mu_K)$ with $K \geq 0$ is a *truncated moment sequence*, that is, whether there is a Radon measure ν, supported on D, such that

$$\int_D y^k \, d\nu(y) = \mu_k, \qquad k = 0, 1, \ldots, K. \tag{1}$$

For $D = [-1, 1]$, $D = (-\infty, \infty)$ and $D = [0, \infty)$ this is referred to as the *truncated Hausdorff moment problem*, the *truncated Hamburger moment problem* and the *truncated Stieltjes moment problem*, respectively. A normalised measure corresponds to $\mu_0 = 1$.

The truncated moment problem has been extensively studied [1, 2, 3]. The set of all truncated moment sequences $(\mu_0, \mu_1, \ldots, \mu_K)$ is the *moment space*; it is a convex subset of $\mathbb{R}^{K+1}$. On the boundary of the moment space the truncated moment problem is determinate; there is a unique representing measure which is always discrete with finitely many atoms. In the interior of the moment space the truncated moment problem is indeterminate; there are infinitely many solutions. An exact characterisation of the boundary for the Hamburger and Stieltjes cases is complicated. We are here ultimately only interested in continuous probability distributions and therefore invoke generic assumptions to simplify matters.

For $L \geq 0$ the $(L+1) \times (L+1)$ Hankel matrices

$$\mathbf{C}_L = (\mu_{i+j})_{i,j=0}^{L}, \tag{2}$$
$$\mathbf{D}_L = (\mu_{i+j} - \mu_{i+j+2})_{i,j=0}^{L}, \tag{3}$$
$$\mathbf{E}_L = (\mu_{i+j} + \mu_{i+j+1})_{i,j=0}^{L}, \tag{4}$$
$$\mathbf{F}_L = (\mu_{i+j} - \mu_{i+j+1})_{i,j=0}^{L}, \tag{5}$$
$$\mathbf{G}_L = (\mu_{i+j+1})_{i,j=0}^{L} \tag{6}$$

are introduced for sequences $(\mu_0, \mu_1, \ldots, \mu_K)$ of sufficient length K and they are assumed to be non-singular. By this prior assumption we only drop solution measures which are finitely atomic. The following statements can be extracted from the literature [1, 2, 3]:

Theorem 1 *Truncated Hausdorff moment problem (Even case: $K = 2M$, Odd case: $K = 2M-1$, $M \geq 1$):*
The sequence $(\mu_0, \mu_1, \ldots, \mu_K)$ is a truncated Hausdorff moment sequence if and only if $\mathbf{C}_M$ and $\mathbf{D}_{M-1}$ for K even (or $\mathbf{E}_{M-1}$ and $\mathbf{F}_{M-1}$ for K odd) are positive definite.

Truncated symmetric Hausdorff moment problem ($K = 2M$, $M \geq 1$, $\mu_{2i-1} = 0$ for $i = 1, \ldots, M$):
The sequence $(\mu_0, 0, \mu_2, \ldots, 0, \mu_K)$ is a truncated Hausdorff moment sequence with a symmetric representing measure if and only if $\mathbf{C}_M$ and $\mathbf{D}_{M-1}$ are positive definite.

Truncated Hamburger moment problem (Even case: $K = 2M$, Odd case: $K = 2M-1$, $M \geq 1$):
The sequence $(\mu_0, \mu_1, \ldots, \mu_K)$ is a truncated Hamburger moment sequence if and only if $\mathbf{C}_M$ for K even (or $\mathbf{C}_{M-1}$ for K odd) is positive definite.

Truncated symmetric Hamburger moment problem ($K = 2M$, $M \geq 1$, $\mu_{2i-1} = 0$ for $i = 1, \ldots, M$):
The sequence $(\mu_0, 0, \mu_2, \ldots, 0, \mu_K)$ is a truncated Hamburger moment sequence with a symmetric representing measure if and only if $\mathbf{C}_M$ is positive definite.

Truncated Stieltjes moment problem (Even case: $K = 2M$, Odd case: $K = 2M - 1$, $M \geq 1$):
The sequence $(\mu_0, \mu_1, \ldots, \mu_K)$ is a truncated Stieltjes moment sequence if and only if $\mathbf{C}_M$ and $\mathbf{G}_{M-1}$ for K even (or $\mathbf{C}_{M-1}$ and $\mathbf{G}_{M-1}$ for K odd) are positive definite.

For $K = 0$ there is a representing measure for all of the truncated moment problems if and only if $\mu_0 > 0$.

For all of the truncated moment problems in the affirmative case there are infinitely many representing measures which are not finitely atomic.

We now distinguish two cases of the truncated moment problem depending on how the sequence $(\mu_0, \mu_1, \ldots, \mu_K)$ is generated: the *statistical* and the *non-statistical* case. A sequence $(\mu_0, \mu_1, \ldots, \mu_K)$ is called a *statistical sequence* if it corresponds to the sample moments of a data sample $\{y_1, \ldots, y_N\}$:

$$\mu_k = \frac{1}{N} \sum_{n=1}^{N} y_n^k, \qquad k = 0, 1, \ldots, K. \tag{7}$$

It is called a *generic statistical sequence* if the underlying data sample contains at least $[K/2] + 1$ distinct data points in $\operatorname{int} D$ where $[K/2]$ is the largest integer smaller or equal $K/2$. To the best knowledge of the author, the following theorem on the statistical case has not been recognised in the literature so far.

Theorem 2 *For $L \geq 0$ the Hankel matrices $\mathbf{C}_L$, $\mathbf{D}_L$, $\mathbf{E}_L$, $\mathbf{F}_L$ and $\mathbf{G}_L$ are positive definite for any statistical sequence $(\mu_0, \mu_1, \ldots, \mu_K)$ of sufficient length K provided the underlying data sample $\{y_1, \ldots, y_N\}$ contains at least $L + 1$ distinct data points in* $\operatorname{int} D$.

For $K \geq 0$ any generic statistical sequence is a truncated moment sequence for the truncated Hausdorff, symmetric Hausdorff, Hamburger, symmetric Hamburger and Stieltjes moment problem, respectively, with a representing measure which is not finitely atomic.

Proof:
For all $L \geq 0$ we observe that for any $\mathbf{a} = (a_0, a_1, \ldots, a_L)^{\mathrm{T}} \in \mathbb{R}^{L+1}$ and $\mathbf{a} \neq 0$

$$\mathbf{a}^{\mathrm{T}} \mathbf{C}_L \mathbf{a} = \frac{1}{N} \sum_{n=1}^{N} P_L^2(y_n) > 0, \tag{8}$$

$$\mathbf{a}^{\mathrm{T}} \mathbf{D}_L \mathbf{a} = \frac{1}{N} \sum_{n=1}^{N} (y_n + 1)(1 - y_n) P_L^2(y_n) > 0, \tag{9}$$

$$\mathbf{a}^{\mathrm{T}}\mathbf{E}_L\mathbf{a} = \frac{1}{N}\sum_{n=1}^{N}(y_n+1)P_L^2(y_n) > 0, \tag{10}$$

$$\mathbf{a}^{\mathrm{T}}\mathbf{F}_L\mathbf{a} = \frac{1}{N}\sum_{n=1}^{N}(1-y_n)P_L^2(y_n) > 0, \tag{11}$$

$$\mathbf{a}^{\mathrm{T}}\mathbf{G}_L\mathbf{a} = \frac{1}{N}\sum_{n=1}^{N}y_nP_L^2(y_n) > 0 \tag{12}$$

with the polynomial

$$P_L(y) = \sum_{i=0}^{L} a_i y^i \tag{13}$$

which can have at most L distinct zeros in $\operatorname{int} D$. Observing the relationships between K and M in Theorem 1 gives the second statement.

In the non-statistical case, the sequence $(\mu_0, \mu_1, \ldots, \mu_K)$ could come from a theory which provides only the moments without reference to a data set and is subject to approximations or errors of any kind (e.g., numerical). Then the positive definiteness of the Hankel matrices and the existence of a solution measure are not guaranteed.

The moment-constrained maximum entropy problem [4, 5, 6] of the first kind seeks the (normalised) probability density function $\hat{p}(y)$ which maximises the Shannon entropy

$$S(p) = -\int_D p(y)\log p(y)\,dy \tag{14}$$

subject to the constraints

$$\int_D \phi_k(y)p(y)\,dy = \mu_k, \qquad k = 1, \ldots, K \tag{15}$$

with prescribed moment functions $\{\phi_1, \ldots, \phi_K\}$ and given moment vector $(\mu_1, \ldots, \mu_K)$. Note that $S(p_X) = S(p) + \log s$. If a solution exists it is unique and has the form of the Gibbs distribution

$$\hat{p}(y) = \exp\left(\lambda_0 + \sum_{k=1}^{K}\lambda_k\phi_k(y)\right) \tag{16}$$

with a set of Lagrangian multipliers $\{\lambda_0, \lambda_1, \ldots, \lambda_K\}$. The parameter λ_0 is determined by normalisation; the other Lagrangian multipliers are found from the moment constraints of eq.(15).

The moment-constrained maximum entropy problem of the second kind seeks the probability density $\hat{p}(y)$ which minimises the relative entropy or Kullback–Leibler divergence with respect to a prescribed reference probability density $\Pi(y)$,

$$I(p|\Pi) = \int_D p(y)\log\frac{p(y)}{\Pi(y)}\,dy \tag{17}$$

subject to the constraints of eq.(15). We have $I(p_X|\Pi_X) = I(p|\Pi)$. If a solution exists it is unique and is given by the Gibbs distribution

$$\hat{p}(y) = \Pi(y)\exp\left(\theta_0 + \sum_{k=1}^{K}\theta_k\phi_k(y)\right) \tag{18}$$

with the Lagrangian multipliers $\{\theta_0, \theta_1, \ldots, \theta_K\}$. The parameter θ_0 is determined by normalisation; the other Lagrangian multipliers are found from the moment constraints of eq.(15).

Again, the statistical and non-statistical cases are distinguished. In the statistical case, maximum likelihood estimation in exponential families under the structural constraint of eq.(2) in the main text without or with a base measure is equivalent to entropy maximisation or relative entropy minimisation, respectively, under the moment constraints of eq.(15); the two problems are convex duals of each other. As such the present work contributes an algorithm for the moment-constrained maximum entropy problem. This is still a topic of discussion [7, 8]; challenges are the lack of orthogonality of polynomial moment functions and the very different scaling of the moment constraints. Both issues are addressed here by the introduction of statistically orthogonal basis functions (see Section 2.5 of the main text). Moreover, the link to the likelihood function allows for a principled selection of moment functions to be included in the moment constraints.

The solutions of the moment-constrained maximum entropy problem of the first kind with polynomial moment functions on the bounded, infinite and semi-infinite domain are subsets of the solutions of the corresponding truncated moment problem. The question arises if there are additional conditions for their existence beyond those stated in Theorem 1 for the truncated moment problems. For the odd case of the truncated Hamburger moment problem the maximum entropy solution generally does not exist. For $K = 0$ the maximum entropy solution is the uniform distribution for the truncated (symmetric) Hausdorff moment problem; it does not exist for the other moment problems. For $K \geq 1$ without prior assumptions on the Hankel matrices the results are as follows ([9, 10, 11] and Theorem 2):

Theorem 3 *Maximum entropy truncated Hausdorff moment problem (Even case: $K = 2M$, odd case: $K = 2M - 1$, $M \geq 1$):*
The maximum entropy solution exists if and only if $\mathbf{C}_M$ and $\mathbf{D}_{M-1}$ for K even (or $\mathbf{E}_{M-1}$ and $\mathbf{F}_{M-1}$ for K odd) are positive definite. It exists for any generic statistical sequence $(\mu_0, \mu_1, \ldots, \mu_K)$.

Maximum entropy truncated symmetric Hausdorff moment problem ($K = 2M$, $M \geq 1$, $\mu_{2i-1} = 0$ for $i = 1, \ldots, M$):
The maximum entropy solution exists if and only if $\mathbf{C}_M$ and $\mathbf{D}_{M-1}$ are positive definite. It exists for any generic statistical sequence $(\mu_0, 0, \mu_2, \ldots, 0, \mu_K)$.

Maximum entropy truncated Hamburger moment problem (Even case: $K = 2M$, $M \geq 1$):
The maximum entropy solution exists if and only if $\mathbf{C}_M$ is positive definite. It exists for any generic statistical sequence $(\mu_0, \mu_1, \ldots, \mu_K)$.

Maximum entropy truncated symmetric Hamburger moment problem ($K = 2M$, $M \geq 1$, $\mu_{2i-1} = 0$ for $i = 1, \ldots, M$):
For $K = 2$ and $K \geq 8$ the maximum entropy solution exists if and only if $\mathbf{C}_M$ is positive definite. It exists for any generic statistical sequence $(\mu_0, 0, \mu_2, \ldots, 0, \mu_K)$.
For $K = 4$ there is additionally the upper bound $\mu_4 \leq 3\mu_2^2/\mu_0$; for $K = 6$ there are additionally the upper bounds $\mu_4 \leq 3\mu_2^2/\mu_0$ and $\mu_6 \leq \Psi(\mu_0, \mu_2, \mu_4)$ where the function Ψ is given in terms of Weber's functions.

Maximum entropy truncated Stieltjes moment problem (Even case: $K = 2M$, odd case: $K = 2M - 1$, $M \geq 1$):
For $K = 1$ and $K \geq 4$ the maximum entropy solution exists if and only if $\mathbf{C}_M$ and $\mathbf{G}_{M-1}$ for K even (or $\mathbf{C}_{M-1}$ and $\mathbf{G}_{M-1}$ for K odd) are positive definite. It exists for any generic statistical sequence $(\mu_0, \mu_1, \ldots, \mu_K)$.

For $K = 2$ there is additionally the upper bound $\mu_2 \leq 2\mu_1^2/\mu_0$; for $K = 3$ there are additionally the upper bounds $\mu_2 \leq 2\mu_1^2/\mu_0$ and $\mu_3 \leq \Lambda(\mu_0, \mu_1, \mu_2)$ where the function Λ is given in terms of Mill's ratio.

The results on the Hausdorff case are in line with those obtained from the regularity of the corresponding exponential families (see S1 Appendix). Here, the interior of the moment space is equal to the set $\operatorname{int}\mathcal{X}$ (see S1 Appendix) and is characterised by the positive definiteness of the relevant Hankel matrices. The results on the Hamburger and Stieltjes cases cannot be immediately obtained from the statistical theory.

The multidimensional truncated moment and moment-constrained maximum entropy problems are less well-studied analytically [3]. But it is worth noting that the multidimensional moment-constrained maximum entropy problem on a bounded domain [8] in the statistical case corresponds to maximum likelihood estimation in a regular exponential family and thus a unique solution generically exists. It is not trivial to quantify exactly how many distinct data points would be required in the data sample for a particular model to guarantee genericity but it is clear that this is never a restriction in practice. Thus any failure to find a solution can only be due to numerical/algorithmic reasons. This fact appears not to be well known as comments in [8] indicate.

References

[1] Shohat JA, Tamarkin JD. The Problem of Moments. American Mathematical Society, Providence, RI; 1963.

[2] Krein MG, Nudelman AA. The Markov Moment Problem and Extremal Problems. American Mathematical Society; 1977.

[3] Schmüdgen K. The Moment Problem. Springer; 2017.

[4] Jaynes ET. Information theory and statistical mechanics. Physical Review. 1957; 106:620–630.

[5] Jaynes ET. On the rationale of maximum-entropy methods. Proceedings of the IEEE. 1982; 70:939–952.

[6] Mead LR, Papanicolaou N. Maximum entropy in the problem of moments. Journal of Mathematical Physics. 1984; 25:2404–2417.

[7] Abramov RV. The multidimensional maximum entropy moment problem: a review on numerical methods. Communications in Mathematical Sciences. 2010; 8:377–392.

[8] Hao W, Harlim J. An equation-by-equation algorithm for solving the multidimensional moment constrained maximum entropy problem. Communications in Applied Mathematics and Computational Science. 2018; 13:189–214.

[9] Frontini M, Tagliani A. Maximum entropy in the finite Stieltjes and Hamburger moment problem. Journal of Mathematical Physics. 1994; 35:6748–6756.

[10] Frontini M, Tagliani A. Entropy-convergence in Stieltjes and Hamburger moment problem. Applied Mathematics and Computation. 1997; 88:39–51.

[11] Tagliani A. Hausdorff moment problem and maximum entropy: a unified approach. Applied Mathematics and Computation. 1999; 105:291–305.
