# Supplementary material for: Semiparametric maximum likelihood probability density estimation
Source: PLoS One. 2021 Nov 9;16(11):e0259111. doi: 10.1371/journal.pone.0259111 (PMC8577774; doi:10.1371/journal.pone.0259111)
Supplement: S3 Appendix — (PDF) [file pone.0259111.s003.pdf]

Supporting Information – S3 Appendix

Semiparametric maximum likelihood probability density estimation

Frank Kwasniok

Alternative methods for density estimation

The alternative density estimators the semiparametric technique is compared against are briefly described.

Kernel density estimation

For a data sample $\{x_1, \ldots, x_N\}$ a kernel density estimator (KDE) [1, 2, 3] is given as

$$\hat{f}_X(x) = \frac{1}{Nh} \sum_{n=1}^{N} K\left(\frac{x - x_n}{h}\right) \tag{1}$$

where the kernel K is a non-negative function on D_X which integrates to unity and $h > 0$ is the bandwidth parameter controlling the degree of smoothing. Here, the Gaussian kernel $K(z) = (1/\sqrt{2\pi}) \exp(-z^2/2)$ is used. The density estimate crucially depends on the choice of bandwidth. Three bandwidth selection methods are considered: firstly, the rule of thumb [1]

$$h_1 = 0.9 \min(\sigma, \mathrm{IQR}/1.34) N^{-1/5} \tag{2}$$

where σ is the sample standard deviation and IQR is the sample interquartile range; secondly, the MATLAB in-built routine `ksdensity` with the default choice of bandwidth; thirdly, the plug-in bandwidth proposed by [4]. These are referred to as KDE1, KDE2 and KDE3, respectively, and the bandwidths are denoted by h_1, h_2 and h_3, respectively. The plug-in bandwidth h_3 is an improvement of the classical Sheather–Jones solve-the-equation rule [5]. It is free of the normal reference rule and is the best available constant bandwidth selection procedure in terms of the mean integrated squared error across a broad range of example densities [4].

A kernel density estimator cannot naturally treat domain boundaries. One can restrict the estimator to the domain and renormalise but experiences a large boundary bias. A log-transform often gives very wiggly density estimates close to the boundary. Boundary correction schemes such as the reflection method or more sophisticated schemes [6] can reduce the boundary bias. We here implement the reflection method on any domain boundary point for all of the kernel density estimators.

The diffusion estimator

The diffusion estimator (DE) [4] starts with the observation that the Gaussian kernel density estimator is linked to solutions of the diffusion partial differential equation or heat equation.

The idea is generalised to processes with state-dependent drift and diffusion. We consider the Fokker–Planck equation

$$\frac{\partial}{\partial t} p_X(x,t) = -\frac{\partial}{\partial x}[A(x)p_X(x,t)] + \frac{1}{2}\frac{\partial^2}{\partial x^2}[B^2(x)p_X(x,t)] \tag{3}$$

on the domain D_X and the associated Itô stochastic differential equation

$$dX_t = A(x)dt + B(x)dW_t \tag{4}$$

where W_t is a standard Wiener process. The drift coefficient is $A(x) = d'(x)/[2p_\mathrm{e}(x)]$ and the diffusion coefficient is $B^2(x) = d(x)/p_\mathrm{e}(x)$ with p_e being a pilot estimate of the probability density f_X and $d(x) = p_\mathrm{e}^\rho(x)$ with $\rho \in [0,1]$ [4]. The Fokker–Planck equation is subject to the reflective boundary condition of zero probability flux, $\frac{\partial}{\partial x}[p_X(x,t)/p_\mathrm{e}(x)] = 0$, at any boundary point. The diffusion estimator can thus handle domain boundaries naturally. Note that the pilot density p_e is the stationary density of the Fokker–Planck equation: $\lim_{t\to\infty} p_X(x,t) = p_\mathrm{e}(x)$.

The Fokker–Planck equation is discretised with finite differences on a bounded interval. For unbounded domains the interval end points are put at $x_\mathrm{a} - 5h_3$ and $x_\mathrm{b} + 5h_3$, respectively, where x_a and x_b are the smallest and largest data point, respectively. For bounded domains $[a, b]$ the interval end points are put at $\max(a, x_\mathrm{a} - 5h_3)$ and $\min(x_\mathrm{b} + 5h_3, b)$, respectively. An equally spaced grid with grid points at the interval end points as well as standard second-order, centred-difference stencils for the derivative of the advective probability flux and the diffusion are used. The reflective boundary conditions at the interval end points are implemented such that the total probability mass as approximated with the trapezoidal quadrature rule on the grid is kept at unity exactly. The resulting system of ODEs is integrated in time with the MATLAB in-built stiff ODE solver `ode15s`.

The density estimate is delivered by integrating the Fokker–Planck equation forward in time from $t = 0$ to $t = t_*$ ($\hat{f}_X(x) = p_X(x, t_*)$) where the initial condition is given by the empirical probability density of the data sample: $p_X(x, 0) = (1/N)\sum_{n=1}^{N} \delta(x - x_n)$. The time t_* plays the role of the bandwidth. It is estimated as [4]

$$t_* = \left(\frac{\mathrm{E}_{f_X}[B^{-1}(X)]}{2\sqrt{\pi}\int_{D_X}(\partial f_X/\partial t)^2\,dx}\frac{1}{N}\right)^{2/5} \tag{5}$$

which minimises the asymptotic mean integrated squared error. Here, $\partial f_X/\partial t$ is the tendency given by the Fokker–Planck equation. We estimate $\mathrm{E}_{f_X}[B^{-1}(X)]$ with the unbiased estimator $(1/N)\sum_{n=1}^{N} B^{-1}(x_n)$. A kernel density estimator with bandwidth rh_3 is used as the pilot density p_e and $\partial f_X/\partial t$ is estimated by inserting the estimator KDE3 into the Fokker–Planck equation. We set $r = 1.5$. This is different from [4] who use KDE3 as the pilot density and estimate $\partial f_X/\partial t$ with the diffusion estimator itself using a bandwidth specially derived to minimise the asymptotic error in this estimate. The method appears to be insensitive to these implementation details; therefore we adopt the simpler procedure. We remark that the practical implementation of the diffusion estimator is not completely straightforward. The pilot density appears in the denominator of both drift and diffusion. If there are regions of almost zero density in long tails or between modes the Fokker–Planck equation produces excessively large tendencies which may cause the ODE integrator to blow up or get stuck as it cannot make progress within the desired accuracy. The problem is compounded by the extreme roughness of the initial condition $p_X(x, 0)$, particularly for high grid resolution. Even in theory, apart from numerical issues, the stability of

the system is not guaranteed [4]. Our choice of a slightly oversmoothed pilot density ($r = 1.5$) appears to mitigate the problem and add some stability to the method. Still, there are test cases of densities for which we were not able to implement the diffusion estimator (see Section 8 of the main text).

We here study the two extreme cases $\rho = 0$ and $\rho = 1$ denoted by DE0 and DE1, respectively. For $\rho = 0$, there is vanishing drift $A(x) = 0$ and the state-dependent diffusion $B(x) = 1/\sqrt{p_\mathrm{e}(x)}$. This is similar to the Gaussian kernel density estimator with variable bandwidth proportional to $1/\sqrt{p_\mathrm{e}(x)}$ [7]. For $\rho = 1$, there is the state-dependent drift $A(x) = p'_\mathrm{e}(x)/[2p_\mathrm{e}(x)]$ and the constant diffusion $B(x) = 1$. This approximately recovers the data sharpening method [8] where the locations of the data points are shifted prior to applying the kernel density estimator. In between ($0 < \rho < 1$) the diffusion estimator resembles a variable location and scale kernel density estimator [9].

Finite mixture models

A finite mixture model [10] represents a probability density as

$$p_X(x) = \sum_{i=1}^{M} \pi_i P(x|\boldsymbol{\theta}_i) \tag{6}$$

with $\pi_i \geq 0$ and $\sum_{i=1}^{M} \pi_i = 1$. The component densities $P(x|\boldsymbol{\theta}_i)$ are members of a standard distribution family on the domain under consideration. We here consider Gaussian, gamma, Weibull and beta mixtures. The parameters of the component densities $\{\boldsymbol{\theta}_i\}_{i=1}^{M}$ and the weights $\{\pi_i\}_{i=1}^{M}$ are estimated via maximum likelihood using the expectation-maximization algorithm [11]. The number of components M is determined with the Bayesian information criterion (BIC).

Local likelihood density estimation

In local likelihood density estimation [12, 13] a simple parametric model for the density f_X is assumed in the neighbourhood of a point x, usually a low-order polynomial model for the log-density,

$$\log p_X(z) = \sum_{i=0}^{M} c_i \left(\frac{z-x}{h}\right)^i \tag{7}$$

with the expansion coefficients $\mathbf{c} = (c_0, c_1, \ldots, c_M)^\mathrm{T}$. The local log-likelihood function is

$$\begin{aligned} l_X(x) &= -N \int_{D_X} K\left(\frac{z-x}{h}\right) p_X(z)\, dz + \sum_{n=1}^{N} K\left(\frac{x_n - x}{h}\right) \log p_X(x_n) \\ &= -N \int_{D_X} K\left(\frac{z-x}{h}\right) \exp\left[\sum_{i=0}^{M} c_i \left(\frac{z-x}{h}\right)^i\right] dz + \sum_{n=1}^{N} K\left(\frac{x_n - x}{h}\right) \sum_{i=0}^{M} c_i \left(\frac{x_n - x}{h}\right)^i \end{aligned} \tag{8}$$

where K is a non-negative kernel function and $h > 0$ is the bandwidth. The estimate of the parameters $\hat{\mathbf{c}} = (\hat{c}_0, \hat{c}_1, \ldots, \hat{c}_M)^\mathrm{T}$ is given by the solution of the local likelihood equations

$$\int_{D_X} K\left(\frac{z-x}{h}\right) \left(\frac{z-x}{h}\right)^i \exp\left[\sum_{i=0}^{M} c_i \left(\frac{z-x}{h}\right)^i\right] dz = \frac{1}{N} \sum_{n=1}^{N} K\left(\frac{x_n - x}{h}\right) \left(\frac{x_n - x}{h}\right)^i \tag{9}$$

for $i = 0, 1, \ldots, M$ and the preliminary density estimate is $\hat{p}_X(x) = \exp(\hat{c}_0)$. Then $\hat{p}_X(x)$ is normalised on D_X to obtain the final density estimate $\hat{f}_X(x)$. The local likelihood density estimator is the local counterpart of a global polynomial exponential family model. The log-likelihood is a strictly concave function of the parameters $\mathbf{c}$. The local likelihood equations match localised population moments with the corresponding localised sample moments. We here consider the cases $M = 0$ (locally constant), $M = 1$ (locally linear) and $M = 2$ (locally quadratic). The estimators are referred to as LLDE0, LLDE1 and LLDE2, respectively. For $M = 0$ the estimator is

$$\hat{p}_X(x) = \frac{\sum_{n=1}^{N} K\left(\frac{x_n - x}{h}\right)}{N \int_{D_X} K\left(\frac{z-x}{h}\right) dz} \tag{10}$$

which recovers the kernel density estimator away from domain boundaries. The standard Gaussian kernel, truncated to the interval $[-5, 5]$, is used. Due to the finite support of the kernel there are no restrictions on the parameters of the locally quadratic model on unbounded domains which allows to model troughs in the density.

For $M = 1$ and $M = 2$, a solution of the local likelihood equations generically uniquely exists and is found with the Newton–Raphson method augmented with a backtracking of the step size to ensure an increase in likelihood at each iteration. The integrals are evaluated using Gauss–Legendre quadrature. If there are very few data points in the interval $[x-5h, x+5h]$ the estimator for $M = 1$ or $M = 2$ may be ill-conditioned or even not exist. At those points we revert back to the locally constant estimator ($M = 0$) which always exists and is stable. If there are no data points in $[x - 5h, x + 5h]$ we have $\hat{p}_X(x) = 0$.

The bandwidth h is determined by 10-fold likelihood cross-validation. The cross-validation likelihood tends to depend rather smoothly on h; therefore it is usually sufficient to maximise it over a relatively coarse grid of bandwidth values.

References

[1] Silverman BW. Density Estimation for Statistics and Data Analysis. Chapman and Hall; 1986.

[2] Wand MP, Jones MC. Kernel Smoothing. Chapman and Hall, London; 1995.

[3] Sheather SJ. Density estimation. Statistical Science. 2004; 19:588–597.

[4] Botev ZI, Grotowski JF, Kroese DP. Kernel density estimation via diffusion. The Annals of Statistics. 2010; 38:2916–2957.

[5] Sheather SJ, Jones MC. A reliable data-based bandwidth selection method for kernel density estimation. Journal of the Royal Statistical Society B. 1991; 53:683–690.

[6] Hall P, Park BU. New methods for bias correction at endpoints and boundaries. The Annals of Statistics. 2002; 30:1460–1479.

[7] Abramson IS. On bandwidth variation in kernel estimates – a square root law. The Annals of Statistics. 1982; 10:1217–1223.

[8] Samiuddin M, El-Sayyad GM. On nonparametric kernel density estimates. Biometrika. 1990; 77:865–874.

[9] Jones MC, McKay IJ, Hu T-C. Variable location and scale kernel density estimation. Annals of the Institute of Statistical Mathematics. 1994; 46:521–535.

[10] McLachlan G, Peel D. Finite Mixture Models. Wiley; 2000.

[11] Dempster AP, Laird NM, Rubin DB. Maximum likelihood from incomplete data via the EM algorithm. Journal of the Royal Statistical Society B. 1977; 39:1–22.

[12] Loader CR. Local likelihood density estimation. The Annals of Statistics. 1996; 24:1602–1618.

[13] Hjort NL, Jones MC. Locally parametric nonparametric density estimation. The Annals of Statistics. 1996; 24:1619–1647.
