# Supplementary material for: Semiparametric maximum likelihood probability density estimation
Source: PLoS One. 2021 Nov 9;16(11):e0259111. doi: 10.1371/journal.pone.0259111 (PMC8577774; doi:10.1371/journal.pone.0259111)
Supplement: S4 Appendix — (PDF) [file pone.0259111.s004.pdf]

Supporting Information – S4 Appendix

Semiparametric maximum likelihood probability density estimation

Frank Kwasniok

Further results

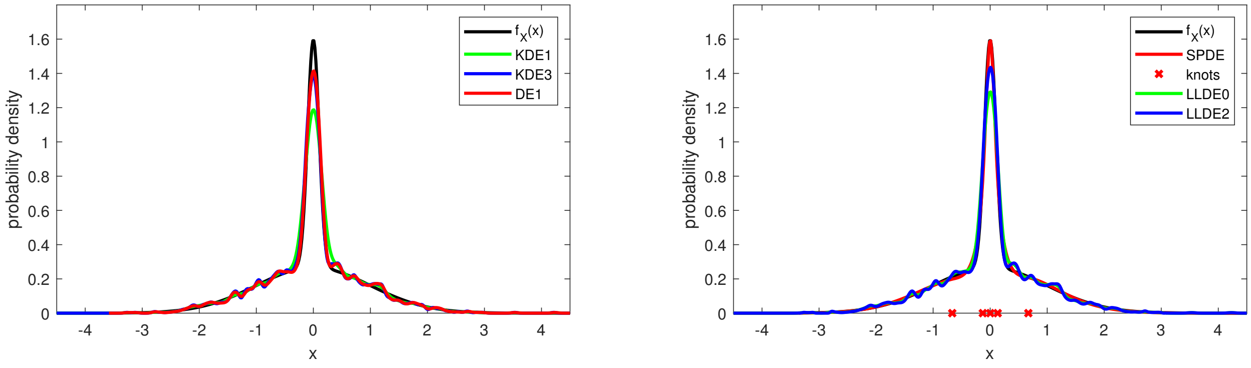

Figure 1: Test case 3: Example of density estimates obtained from the kernel density and diffusion estimators (left) as well as from the semiparametric and local likelihood estimators (right). The SPDE is the model SPL2 with 5 knots after knot deletion. The sample size is $N = 2000$.

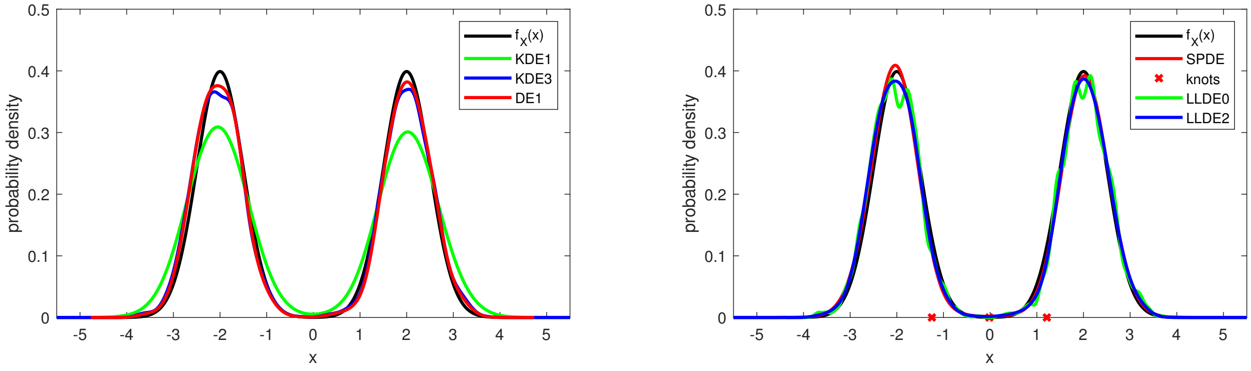

Figure 2: Test case 7: Example of density estimates obtained from the kernel density and diffusion estimators (left) as well as from the semiparametric and local likelihood estimators (right). The SPDE is the model SPL1 with 3 knots after knot deletion. The sample size is $N = 2000$.

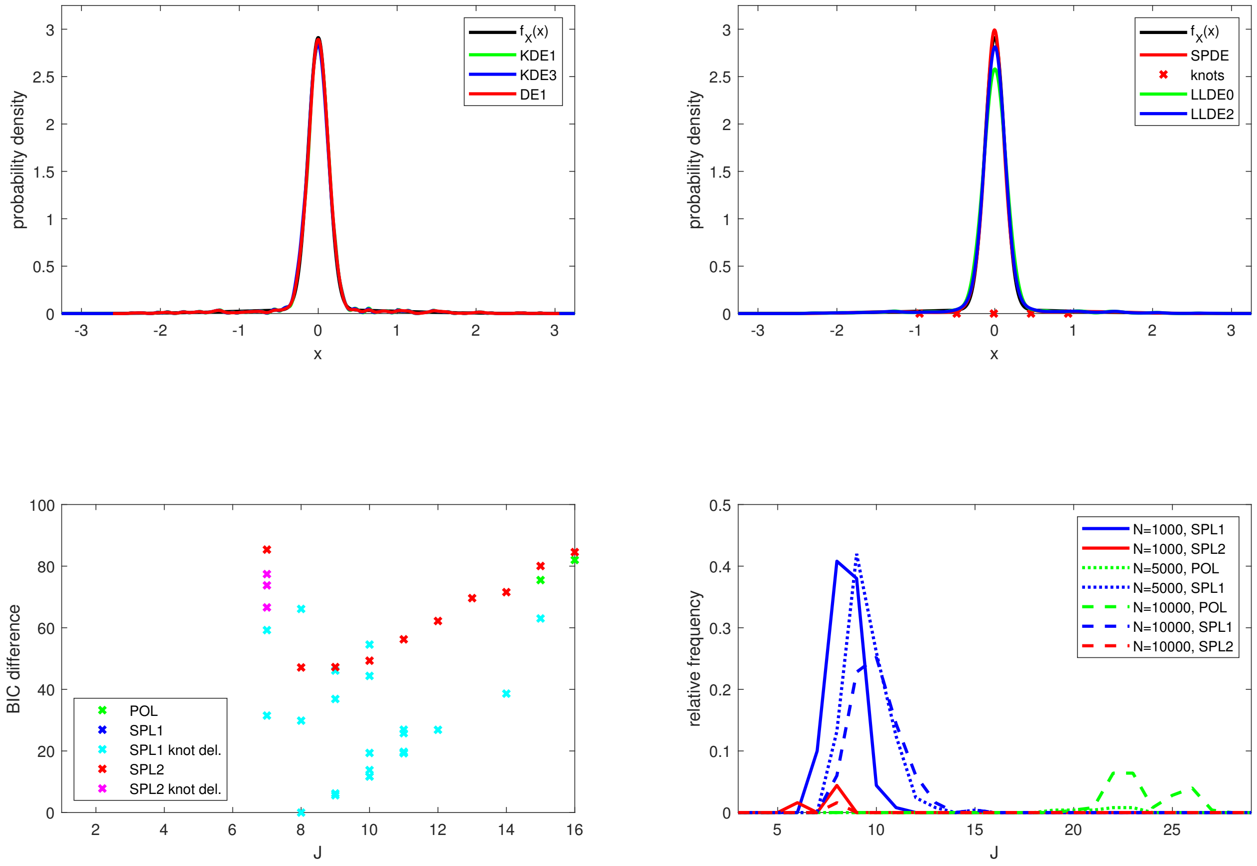

Figure 3: Test case 4: Example of density estimates obtained from the kernel density and diffusion estimators (top left) as well as from the semiparametric and local likelihood estimators (top right). The SPDE is the model SPL1 with 5 knots after knot deletion. The sample size is $N = 2000$. Bottom left: Corresponding model selection. Bottom right: Relative frequency out of 250 realisations of picking a particular model as the SPDE. The spline models encompass models without and with knot deletion.

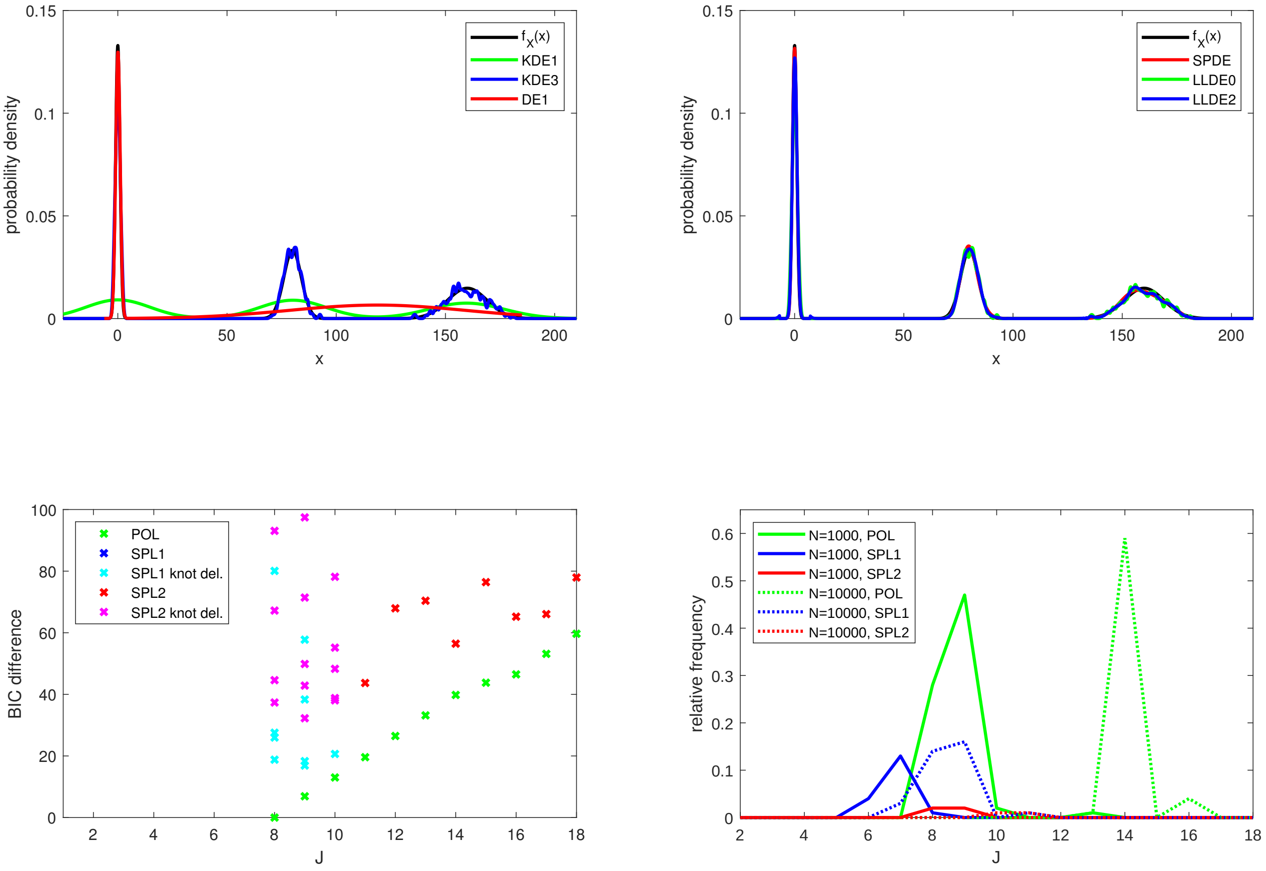

Figure 4: Test case 9: Example of density estimates obtained from the kernel density and diffusion estimators (top left) as well as from the semiparametric and local likelihood estimators (top right). The SPDE is the model POL of 8th order. The sample size is $N = 1000$. Bottom left: Corresponding model selection. Bottom right: Relative frequency out of 250 realisations of picking a particular model as the SPDE. The spline models encompass models without and with knot deletion.

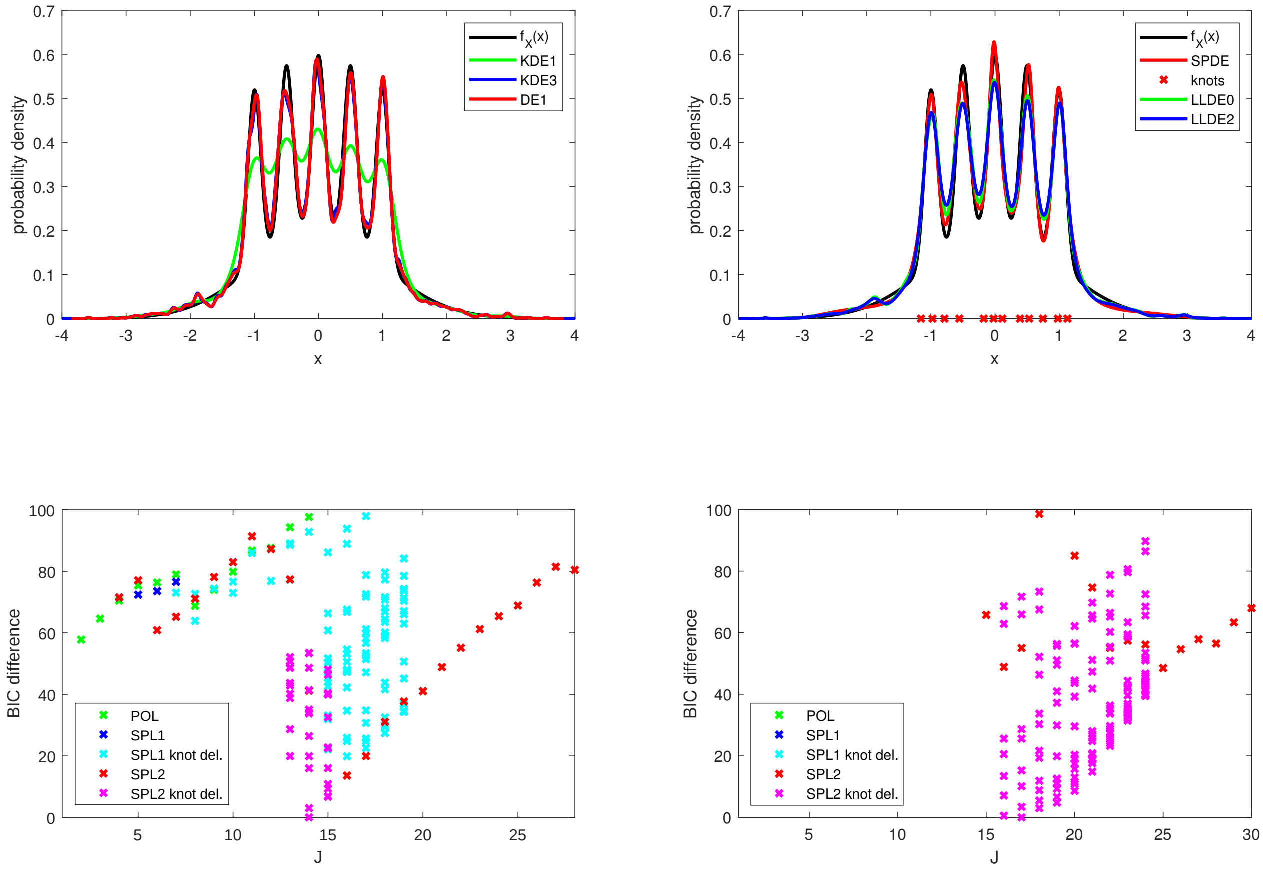

Figure 5: Test case 11: Example of density estimates obtained from the kernel density and diffusion estimators (top left) as well as from the semiparametric and local likelihood estimators (top right). The SPDE is the model SPL2 with 12 knots after knot deletion. The sample size is $N = 5000$. Bottom left: Example of model selection for $N = 1000$. Bottom right: Example of model selection for $N = 10000$.
